# Cerebrospinal fluid and positron-emission tomography biomarkers for noradrenergic dysfunction in neurodegenerative diseases: a systematic review and meta-analysis

**DOI:** 10.1093/braincomms/fcad085

**Published:** 2023-03-29

**Authors:** Elisa Lancini, Lena Haag, Franziska Bartl, Maren Rühling, Nicholas J Ashton, Henrik Zetterberg, Emrah Düzel, Dorothea Hämmerer, Matthew J Betts

**Affiliations:** German Center for Neurodegenerative Diseases (DZNE), Otto-von-Guericke University Magdeburg, Magdeburg, Germany; Faculty of Medicine, Institute of Cognitive Neurology and Dementia Research (IKND), Otto-von-Guericke University Magdeburg, Magdeburg, Germany; German Center for Neurodegenerative Diseases (DZNE), Otto-von-Guericke University Magdeburg, Magdeburg, Germany; Faculty of Medicine, Institute of Cognitive Neurology and Dementia Research (IKND), Otto-von-Guericke University Magdeburg, Magdeburg, Germany; Faculty of Medicine, Institute of Cognitive Neurology and Dementia Research (IKND), Otto-von-Guericke University Magdeburg, Magdeburg, Germany; Faculty of Medicine, Institute of Cognitive Neurology and Dementia Research (IKND), Otto-von-Guericke University Magdeburg, Magdeburg, Germany; Institute of Psychiatry, Department of Old Age Psychiatry, King’s College London, London, UK; Wallenberg Centre for Molecular and Translational Medicine, University of Gothenburg, Gothenburg, Sweden; NIHR Biomedical Research Centre for Mental Health & Biomedical Research Unit for Dementia at South London & Maudsley NHS Foundation, London, UK; Department of Psychiatry and Neurochemistry, Institute of Neuroscience & Physiology, the Sahlgrenska Academy at the University of Gothenburg, Mölndal, Sweden; Department of Psychiatry and Neurochemistry, the Sahlgrenska Academy at the University of Gothenburg, Mölndal, Sweden; Clinical Neurochemistry Laboratory, Sahlgrenska University Hospital, Mölndal, Sweden; Department of Neurodegenerative Disease, UCL Institute of Neurology, London, UK; UK Dementia Research Institute at UCL, London, UK; Hong Kong Center for Neurodegenerative Diseases, Hong Kong, China; German Center for Neurodegenerative Diseases (DZNE), Otto-von-Guericke University Magdeburg, Magdeburg, Germany; Faculty of Medicine, Institute of Cognitive Neurology and Dementia Research (IKND), Otto-von-Guericke University Magdeburg, Magdeburg, Germany; Institute of Cognitive Neuroscience, University College London, London, UK; Center for Behavioral Brain Sciences, University of Magdeburg, Magdeburg, Germany; German Center for Neurodegenerative Diseases (DZNE), Otto-von-Guericke University Magdeburg, Magdeburg, Germany; Faculty of Medicine, Institute of Cognitive Neurology and Dementia Research (IKND), Otto-von-Guericke University Magdeburg, Magdeburg, Germany; Institute of Cognitive Neuroscience, University College London, London, UK; Center for Behavioral Brain Sciences, University of Magdeburg, Magdeburg, Germany; Department of Psychology, University of Innsbruck, Innsbruck, Austria; German Center for Neurodegenerative Diseases (DZNE), Otto-von-Guericke University Magdeburg, Magdeburg, Germany; Faculty of Medicine, Institute of Cognitive Neurology and Dementia Research (IKND), Otto-von-Guericke University Magdeburg, Magdeburg, Germany; Center for Behavioral Brain Sciences, University of Magdeburg, Magdeburg, Germany

**Keywords:** noradrenaline, norepinephrine, Alzheimer’s disease, Parkinson’s disease, locus coeruleus

## Abstract

The noradrenergic system shows pathological modifications in aging and neurodegenerative diseases and undergoes substantial neuronal loss in Alzheimer’s disease and Parkinson’s disease. While a coherent picture of structural decline in post-mortem and *in vivo* MRI measures seems to emerge, whether this translates into a consistent decline in available noradrenaline levels is unclear.

We conducted a meta-analysis of noradrenergic differences in Alzheimer’s disease dementia and Parkinson’s disease using CSF and PET biomarkers.

CSF noradrenaline and 3-methoxy-4-hydroxyphenylglycol levels as well as noradrenaline transporters availability, measured with PET, were summarized from 26 articles using a random-effects model meta-analysis.

Compared to controls, individuals with Parkinson’s disease showed significantly decreased levels of CSF noradrenaline and 3-methoxy-4-hydroxyphenylglycol, as well as noradrenaline transporters availability in the hypothalamus. In Alzheimer’s disease dementia, 3-methoxy-4-hydroxyphenylglycol but not noradrenaline levels were increased compared to controls.

Both CSF and PET biomarkers of noradrenergic dysfunction reveal significant alterations in Parkinson’s disease and Alzheimer’s disease dementia. However, further studies are required to understand how these biomarkers are associated to the clinical symptoms and pathology.

## Teaser

Lancini *et al.* showed that compared to controls, CSF and PET noradrenergic biomarkers are decreased in Parkinson’s disease, while in Alzheimer’s disease, only CSF noradrenaline metabolite 3-methoxy-4-hydroxyphenylglycol levels are significantly increased.

Further studies should determine how CSF measures of noradrenergic dysfunction are related to pathology and clinical symptoms.

## Graphical Abstract

**Graphical abstract:**
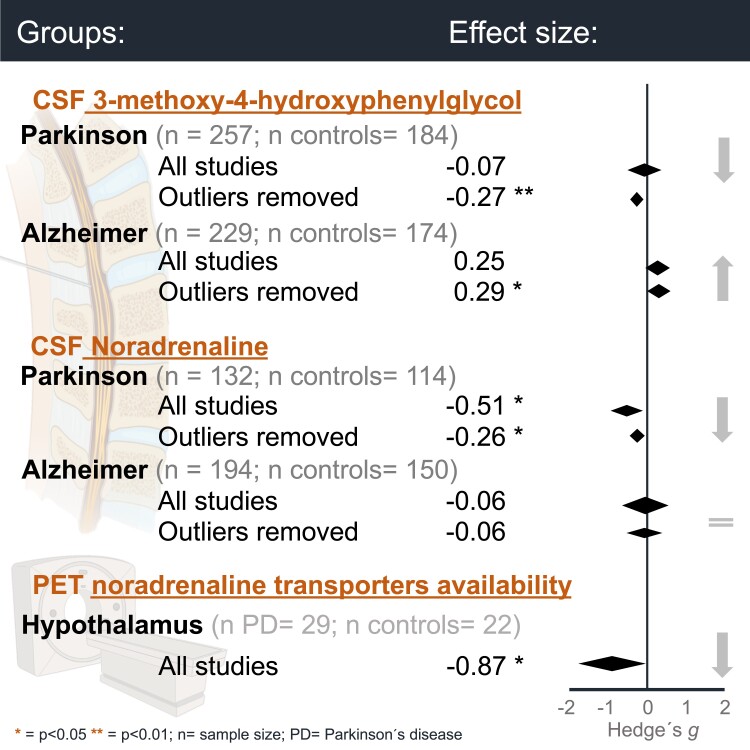

## Introduction

Pathological alterations to the locus coeruleus (LC), a major source of noradrenaline (NA) in the brain, occur early in Alzheimer’s and Parkinson’s disease.^1,2^ While degeneration in the LC can influence the function of other brain areas directly via noradrenergic dysregulation and related cognitive changes,^3^ it can also lead to increased neuroinflammation and tau propagation, thereby likely contributing to neurodegeneration in Alzheimer’s and Parkinson’s disease.^4–6^ Given the involvement of the noradrenergic system in neurodegenerative diseases, noradrenergic biomarkers could be an important complementary tool to established pathological biomarkers and may provide new insights into the neuromodulatory underpinnings of cognitive and behavioural symptoms. As a consequence, MRI techniques that allow to characterize the integrity of the LC *in vivo*^7^ have attracted a lot of interest in recent years. Together with post-mortem evidence, substantial degeneration to the LC has been observed in Alzheimer’s and Parkinson’s disease.

In Alzheimer’s disease, tau accumulation in the LC precedes volume loss, with a decrease of more than 55% of LC neurons during the progression from prodromal to severe dementia.^8,9^ Neuronal loss may be more prominent in the rostral/middle portion of the LC,^9,10^ and has been shown to correlate with decreased cognitive function,^11–13^ post-mortem neuropathology^8^ and reduced NA levels in the neocortex and hippocampus.^14^

In Parkinson’s disease and synucleinopathies, a-synuclein containing Lewy bodies and neuronal cell loss in the LC^9,15^ may affect NA synthesis^16^ and precede degeneration to the substantia nigra.^1,17,18^ A number of different clinical features related to noradrenergic dysfunction have been observed in Parkinson’s disease,^19^ particularly non-motor symptoms^20^ that may precede motor symptomatology and become more prevalent with disease progression.^21^

Clinical features related to noradrenergic dysfunction, such as anxiety and depression, are risk factors for Alzheimer’s disease and may underlie common non-motor symptoms observed in Parkinson’s disease, and cognitive functions most affected in aging depend in part on the noradrenergic system.

A variety of studies found a relation between age-related noradrenergic system decline and episodic memory decline as well as lower cognitive reserve in normal ageing as measured by structural imaging^22–25^ and post-mortem assays.^8,13^ Corroborating this link between cognitive function and LC degeneration, a recent study suggested that maintaining the neural density of the LC–NA nuclei may prevent cognitive decline in aging.^13,26^ Furthermore, early clinical symptoms of neurodegenerative diseases may originate from noradrenergic dysfunction, such as sleep-wake cycle dysregulation, depression, anxiety, agitation,^5,27–30^ impaired attention and memory,^4^ suggesting that the integrity of the LC–NA system may be critical for tracking the progression from healthy to pathological aging.^31^

Taking into account all of the above, noradrenergic dysfunction occurs in healthy aging and is amongst the earliest signs of Alzheimer’s and Parkinson’s-like neuropathology. Therefore, monitoring the status of the noradrenergic system and levels of NA and its metabolites may be informative for understanding the neural underpinnings of clinical symptoms and assessment of disease progression.

While a structural decline in LC seems to emerge as a consistent phenomenon early on in Alzheimer’s and Parkinson’s disease, it is unclear how this relates to NA availability. Moreover, post-mortem and *in vivo* studies indicate that NA output might be upregulated potentially as a compensatory mechanism following structural decline in the LC,^32–36^ thus assessments of NA availability may help to understand these effects further.

In this meta-analysis and systematic review, we aimed to investigate the extent to which NA levels are affected in Alzheimer’s and Parkinson’s disease using CSF and PET markers of noradrenergic dysfunction. We review studies reporting CSF measures of NA and its major metabolite 3-methoxy-4-hydroxyphenylglycol (MHPG)^37^ and those using the PET radioligand 11C-MeNER (MeNER) that binds to NA transporters (NATs), as a biomarker of NA availability in the brain. We also aimed to assess to what extent NA levels are dependent on disease severity and contrasting analytical techniques.

## Materials and methods

### Search strategy, selection criteria and included studies

We searched PubMed for relevant English articles. Four authors (E.L., M.R., L.H and F.B.) independently extracted the relevant information and individually assessed the quality of the evidence with *robvis* online bias tool (Supplementary Fig. 1).^38^ In case of missing data, corresponding authors were contacted and if no additional information was provided, data points were extrapolated from the article’s plots using the online software WebPlotDigitalizer Version 4.4.^39^ Reviews and articles with previously published or unpublished data were excluded (see Supplementary Methods for more details).

**Figure 1:**
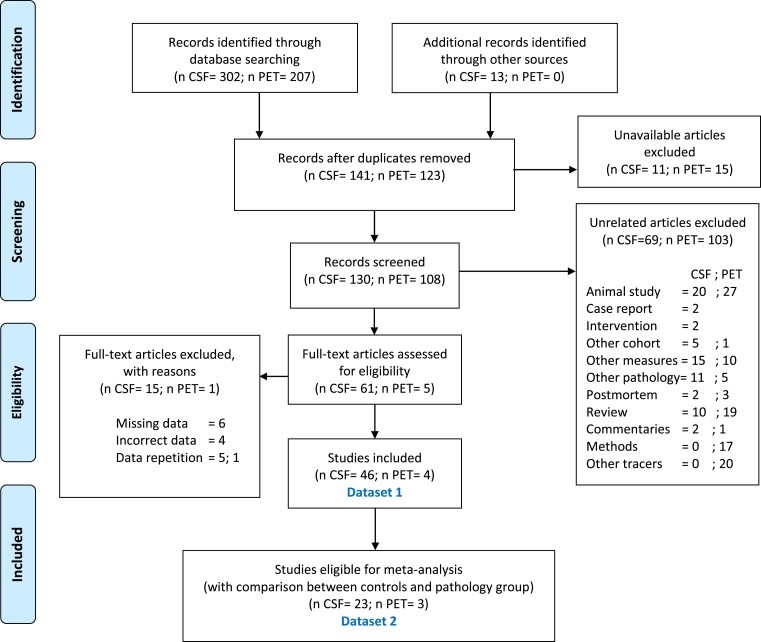
**Flow diagram representing articles selection and inclusion process according to the PRISMA guidelines.** The data collected for this review and meta-analysis is divided into two separate datasets. ‘Dataset 1’ was composed of 50 studies (CSF *k* = 46, PET *k* = 4). Of those articles, 26 studies (CSF *k* = 23, PET *k* = 3) report comparisons between controls and Alzheimer’s disease-type dementia (*k* = 15 for CSF) and/or Parkinson’s disease (*k* = 13 for CSF, *k* = 3 for PET), referred to here as *dataset 2*. Information on all articles included in both groups can be found in Supplementary Table 1. Numbers of CSF and PET articles, respectively, are divided by a semi column. Flow Diagram adapted from: Moher *et al.*^43^. For more information, visit www.prisma-statement.org.

Throughout the paper, we refer to the study participants using the umbrella term Alzheimer’s disease-type dementia (ADD) since the majority of studies reported did not confirm presence of Alzheimer’s disease pathology. Additionally, PD refers to idiopathic Parkinson’s disease and individuals with Parkinson’s disease dementia. Among the studies included in the meta-analysis, only *k* = 3^40–42^ of *k* = 15 studies comparing between Alzheimer’s disease-type dementia clinical group and controls (CONTR) assessed Alzheimer’s disease-type dementia pathology using amyloid (*k* = 2^41,42^), phospho-tau (*k* = 3^40–42^) and total-tau (*k* = 2^41,42^); therefore, the absence of Alzheimer’s disease pathological measures was not used as an exclusion criterion. A detailed flow diagram of the literature search and exclusion criteria is shown in Fig. 1.

A total of 50 studies (CSF *k* = 46, PET *k* = 4) reported adequate data (mean and standard deviation) CSF MHPG, CSF NA, or PET MeNER data for individuals with Alzheimer’s disease-type dementia, Parkinson’s disease and/or controls, or it was possible to extrapolate it from plots or calculate it from median data. We refer to this group of articles and related data as *dataset 1*. Of those articles, 26 studies (CSF *k* = 13, PET *k* = 3) reported a suitable comparison between controls and Alzheimer’s disease-type dementia (*k* = 15 for CSF) and/or Parkinson’s disease (*k* = 13 for CSF, *k* = 3 for PET) and adequate data (average and standard deviation) for the calculation of the meta-analysis, or it was possible to extrapolate it from plots or calculate it from median data. We will refer to this subgroup, in which the analysis of the effect size and thus the meta-analysis was possible, as *dataset 2*. No studies using PET MeNER in Alzheimer’s disease-type dementia were found. All articles are included in a qualitative synthesis in Supplementary Table 1.

Apart from the mean and standard deviations (SD) for each study, in *dataset 1* we also noted data concerning other informative variables, namely sample size (*n*), analytical method used to evaluate the noradrenergic levels of CSF (‘method’), volume of the CSF sample (‘csfvol’), age (‘age’), years post diagnosis (‘ypd’), and disease severity (‘severity’), based on the Hoehn and Yahr (H&Y) scores for Parkinson’s disease group (mild = 1–2; moderate = 3; severe = 4–5) and Mini-Mental State Examination (MMSE) scores for Alzheimer’ dementia group (normal >24; mild = 21–24; moderate = 13–20; severe: < 12), only if reported for the same number of participants who provided CSF data, with an accepted 5-participant deviation range.

This review was performed according to the preferred reporting items for systematic reviews and meta-analyses guidelines (PRISMA).^43^ Further details on the search strategy, review criteria and data extraction can be found in the Supplementary material. The review was not preregistered.

### Random-effect mixed-model meta-analysis assessing group differences in noradrenaline levels across studies (‘dataset 2’)

Statistical analyses were carried out using R software (version R i386 3.4.2).^44^ Only articles reporting comparisons between either Alzheimer’s disease-type dementia or Parkinson’s disease and a control group were included in the meta-analyses. For every article, independent Welch’s *t*-test was conducted to assess mean differences between control and Parkinson’s disease or Alzheimer’s disease-type dementia groups. Then, the standardized mean differences (SMD) were calculated with Hedge’s *g*, Cohen’s *d* effect size corrected for small samples.^45^ Finally, we performed a random-effect mixed-model meta-analysis using the ‘metagen’ function from the R package meta.^46^

The levels of CSF NA and CSF MHPG were investigated in both Alzheimer’s disease-type dementia (ADD NA and ADD MHPG) and Parkinson’s disease (PD NA and PD MHPG), compared to controls. Given limited data availability, the levels of MeNER PET were measured only in Parkinson’s disease compared to controls and only in the hypothalamus, LC, median raphe, nucleus ruber and thalamus. Although some articles included additional regions, only those mentioned in more than one paper can be potentially included in a meta-analysis. The estimation of the average true effect (μ) was calculated with a 95% confidence interval (CI) and the between-study-variance using the tau-squared estimator (τ2). The heterogeneity across studies is a recognized issue in meta analyses.^47^ To adjust for heterogeneity across studies i.e. differing sample sizes, the adjustment method of the CI proposed by Hartung–Knapp–Sidik–Jonkman was used for the calculation of the CI of the pooled effect size. Additionally, (i) statistical outliers (studies whose 95% CI of effect sizes lies outside the 95% CI of the pooled effect) were identified using the R function ‘find.outliers’ and (ii) potential influential cases (studies whose exclusion from the analysis led to significant changes in the fitted model^46^ as shown in Supplementary Fig. 2) were removed from subsequent analyses. Regarding CSF measures, a total of five studies (*k* = 5) were identified as outliers or influential cases and subsequently removed from further analyses (ADD NA: *k* = 2; PD NA: *k* = 2, ADD MHPG: *k* = 3; PD MHPG: *k* = 1, see also Supplementary Table 2). The meta-analysis model was then re-calculated excluding the detected outliers and influential cases, as evaluated by a leave-one-out approach.^48^ In the PET studies, no outliers were identified, and analysis of influential cases was not possible due to the small number of studies per brain region (*k* = 2).

### Stepwise regression analyses to assess for inter-study heterogeneity (‘dataset 1’)

To assess for a potential influence of inter-study differences in measurement methods and sample characteristics on the meta-analyses results, stepwise regression analyses were conducted using the mean values reported from studies in ‘dataset 1’. Investigated variables in these regressions were (i) study-related confounds (sample size, analytical method used to evaluate the noradrenergic levels of CSF and volume of the CSF sample) and where data were available, (ii) variables assumed to influence NA and MHPG levels within study groups (age, years post diagnosis and disease severity).

Regressions on ‘dataset 2’ data were first conducted separately for each clinical group and noradrenergic outcome measure, resulting in six separate regressions (ADD MHPG, ADD NA, PD MHPG, PD NA, CONTR MHPG and CONTR NA, model: ~*n* +‘method’ + ‘csfvol’ + ‘age’ *+* ‘severity’ + ‘ypd’). Within each group and noradrenergic measure, the number of studies reporting each regressor were assessed (ADD MHPG: ‘method’ *k* = 25; ‘csfvol’ *k* = 20; ‘age’ *k* = 15, ‘severity’ *k* = 4, ‘ypd’ *k* = 6; ADD NA: ‘method’ *k* = 16, ‘csfvol’ *k* = 15, ‘age’ *k* = 14, ‘severity’ *k* = 9, ‘ypd’ *k* = 5, CONTR NA: ‘method’ *k* = 16, ‘csfvol’ *k* = 14, ‘age’ *k* = 15; PD MHPG: ‘method’ *k* = 28, ‘csfvol’ *k* = 22, ‘age’ *k* = 18, ‘severity’ *k* = 7, ‘ypd’ *k* = 10; PD NA: ‘method’ *k* = 18, ‘csfvol’ *k* = 17, ‘age’ *k* = 11, ‘severity’ *k* = 8, ‘ypd’ *k* = 8; CONTR NA: ‘method’ *k* = 22, ‘csfvol’ *k* = 18, ‘ age’ *k* = 21). If data for a particular regressor were not available for at least half of the studies within each group, the regressor was removed from the regression models for that group. See Supplementary Table 2 for an overview of included regressors. In the included regressors, missing values were replaced with the mean value of the variable. The variables were centred and *z*-scored in order to allow comparison between variables measured on different scales.

As there were six different methods and three groups, the variables ‘method’ and ‘ group’ were summarized into factors using the ‘as.factor()’ function (see Supplementary Fig. 3 for an example of the workflow on the ‘dataset 1’—MHPG). All the other variables were treated as continuous and entered as single regressor variables. For the control groups (CONTR), the variables ‘severity’ and ‘ypd’ were removed from the model as these were not applicable. Brain pathology, as indicated by measures of CSF amyloid and tau, could not be included as a regressor as they were only reported in a few studies (*k* = 3^40–42^).

Secondly, stepwise analysis was performed on the data collapsed across groups and therefore divided only with respect to the noradrenergic measures (NA and MHPG). These included the variable ‘ group’ (AD, PD and CONTR) as a factor to control for known group mean differences: (model: ~*n* + ‘method’ + ‘csfvol’ + ‘group’ + ‘age’ + ‘severity’ + ‘ypd’) as noted in Supplementary Table 5. Stepwise regressions were performed using the R function ‘stepAIC()’, option direction ‘both’, that selects the most contributing regressors and removes those who do not improve the model fit, using Akaike information criterion (AIC).^49^ Each continuous variable was entered as a single regressor. The levels (*W*) of each factor variables ‘method’ and ‘group’, were dummy coded using as a reference level ‘HPLC’ and ‘CONTR’, respectively, resulting in *W*-1 regressors each (see Supplementary Fig. 3 for a detailed description).

Additional analyses using weighted means of ‘dataset 1’ can be found in the Supplementary Material (Supplementary Table 3, Supplementary Fig. 4 and Fig. 5).

### Data availability

The code for all analyses described is available and can be downloaded at https://github.com/ElisaLancini/meta-analysis.

## Results

The data collected for this review and meta-analysis are divided into two separate datasets. We found a total of 50 studies (CSF *k* = 46, PET *k* = 4), referred to as ‘dataset 1’. Of these, 26 reported differences in Alzheimer’s disease-type dementia (*k* = 15) and Parkinson’s disease (*k* = 13 for CSF, *k* = 3 for PET) compared to controls, and compose the ‘dataset 2’.

Based on established interpretations of effect size magnitudes,^50^ we generally obtained small effects for the CSF meta-analysis comparing CSF NA and MHPG levels between either Alzheimer’s disease-type dementia/Parkinson’s disease compared to controls (*d* = 0.2), whilst for the PET meta-analysis, comparing NATs density levels between Parkinson’s disease and controls, effect sizes were in the medium (*d* = 0.5) to large (*d* = 0.9) range. A positive effect size indicates higher levels in clinical groups compared to controls, whilst a negative effect size indicates lower levels compared to controls.

**Figure 2:**
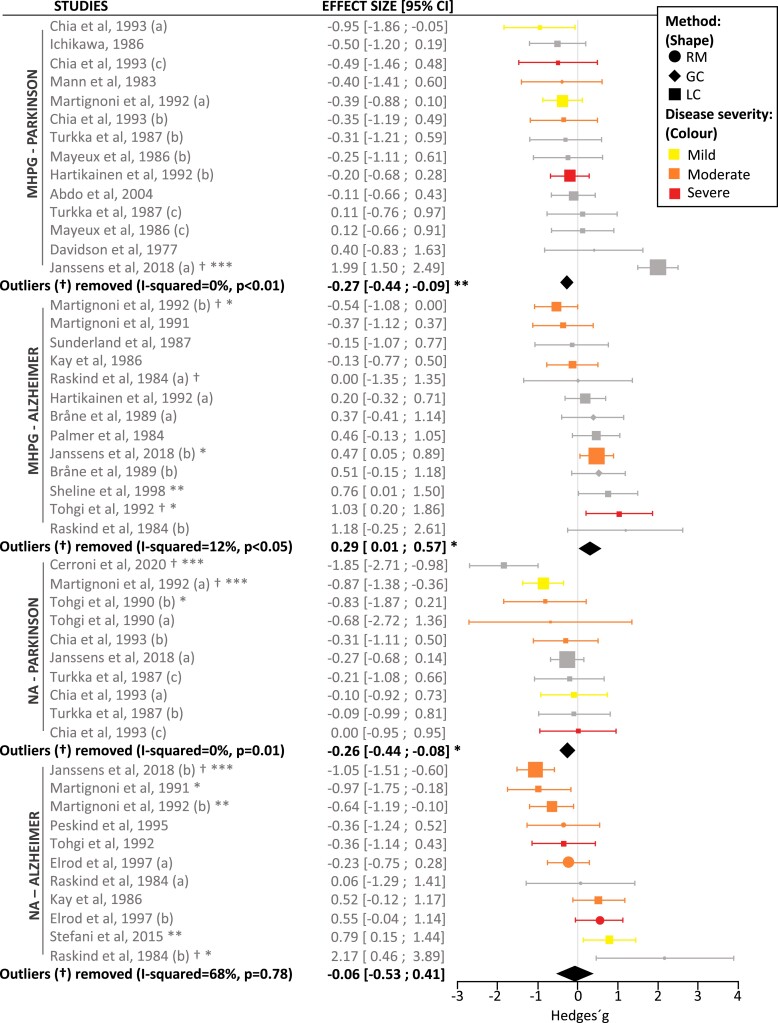
**Meta-analysis results of NA and MHPG levels in CSF.** The forest plot shows the effect sizes between Alzheimer’s disease-type dementia and Parkinson’s disease compared to controls. The averaged effect size and 95% CI is indicated by the black diamonds. The size of the symbols indicates the pooled number of participants in each study. Significance levels are indicated by asterisks (*P < 0.05, ** *P* < 0.01, ***P < 0.001). The significance of a single study refers to the result of the Welch’s *t*-test between the means of the two groups analysed. Studies excluded as outliers are indicated with the symbol †. The studies were characterized on the basis of the analytical method used to evaluate CSF NA and MHPG, as illustrated using different-shaped data points, where symptom severity was also differentially illustrated using different coloured data points. Clinical severity was based on H&Y scores for Parkinson’s disease group (mild = 1–2; moderate = 3; severe = 4–5) and MMSE scores for Alzheimer’ dementia group (normal >24; mild = 21–24; moderate = 13–20; severe: < 12). GC = gas chromatography; H&Y = Hoehn and Yahr scale; LC = liquid chromatography; MHPG = 3-methoxy-4-hydroxyphenylglycol; MMSE = mini-mental state examination; NA = noradrenaline; RM = radioenzymatic methods.

**Figure 3:**
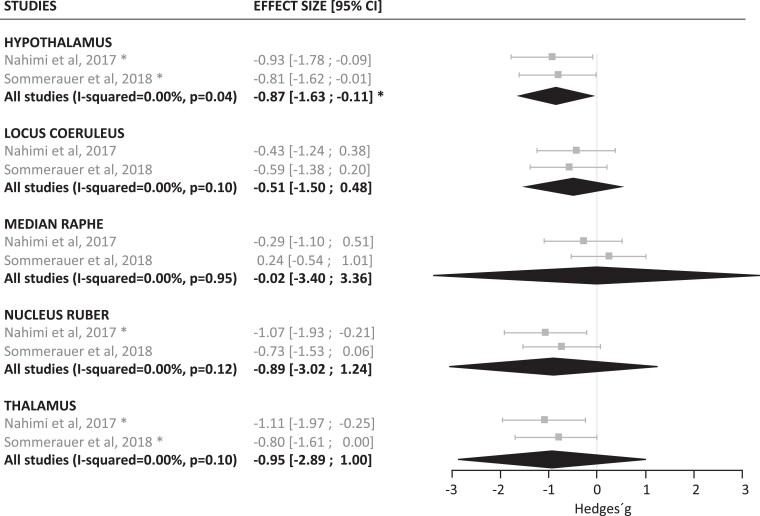
**Meta-analysis results of PET MeNER binding in Parkinson’s disease and control groups.** The forest plot shows the effect sizes of the disease group compared to controls. The averaged effect size and 95% CI is indicated by the black diamonds. The application of the Hartung–Knapp–Sidik–Jonkman method (HKSJ) results in more conservative CI, which might exceed the variance of the single studies when the number of included studies is small and when standard errors vary considerably between them. The size of the symbols indicates the pooled number of participants in each study. Significance levels are indicated by asterisks (*P < 0.05, ** *P* < 0.01, ***P < 0.001) for summarized as well as individual studies. The significance of a single study refers to the result of the Welch’s *t*-test between the averages of the two groups analysed. CI = confidence interval; MeNER = (S, S)-11C-2-(a-(2-methoxyphenoxy)benzyl)morpholine.

In line with degeneration to the noradrenergic system, a significant reduction in CSF NA (*n* = 132, *g* = −0.26, *P* = 0.01), CSF MHPG (*n* = 257, *g* = −0.27, *P* = 0.006) (Fig. 2) as well as PET MeNER binding in the hypothalamus (*n* = 29, *g* = −0.87, *P* < 0.05), was observed in Parkinson’s disease compared with control subjects (*n* = 114 for NA, *n* = 184 for MHPG and *n* = 22 for PET, respectively) (Fig. 3). In the PET MeNER meta-analysis, other brain regions such as the LC (*n* = 22, *g* = −0.51, *P* = 0.10), median raphe (*n* = 22, *g* = −0.02, *P* = 0.95), nucleus ruber (*n* = 22, *g* = −0.89, *P* = 0.12) and thalamus (*n* = 22, *g* = −0.95, *P* = 0.10) did not differ significantly in NATs density levels compared to controls. Exclusion of Parkinson’s disease studies considered outliers from the CSF analysis reduced the between-study heterogeneity (I-squared) from 45.05% to 0.00% for NA and from 82.81% to 0.00% for MHPG (Supplementary Table 2), leading to a change in the *P*-value of the pooled effect size from 0.019 to 0.012 for NA and from 0.733 to 0.006 for MHPG (Supplementary Table 2) following exclusion of *k* = 2 and *k* = 1 studies, respectively.

In Alzheimer’s disease-type dementia, no significant difference in CSF NA levels (*n* = 194, *g* = −0.06, *P* = 0.78) was found, however a significant, yet small increase in CSF MHPG (*n* = 229, *g* = 0.29, *P* = 0.04) was observed compared to controls (*n* = 150 for NA, *n* = 174 for MHPG, respectively). No *in vivo* studies using PET MeNER in Alzheimer’s disease were found, therefore no meta-analysis was conducted for this clinical group. Exclusion of outliers from the CSF analysis reduced the *I*-squared from 78.92% to 67.87% for NA, and from 45.73% to 11.99% for MHPG (Supplementary Table 2), leading to a change in the *P*-value from 0.825 to 0.778 for NA and from 0.090 to 0.042 for MHPG, following exclusion of *k* = 2 and *k* = 3 studies, respectively (Supplementary Table 2).

Among the regression models, none of the full models were significant (see Supplementary Table 4).

Significant reduced models were found for Parkinson’s disease MHPG (*P* = 0.018, Supplementary Table 5), as well as the regressions on MHPG and NA combined across groups (*P* = 0.003; *P* = 0.032, Supplementary Table 5). However, ANOVA analyses did not show any significant improvement in explained variance when the reduced models were compared to the full models (see Supplementary Table 6).

A trend towards significance was observed in the Alzheimer’s disease-type dementia NA and Parkinson’s disease NA group (*P* = 0.058; *P* = 0.059, Supplementary Table 5).

The results of the fitted reduced regression models obtained (Supplementary Table 5) taking each clinical group and noradrenergic outcome measure showed that larger sample sizes were related to higher CSF NA in Alzheimer’s disease-type dementia (*t* = 2.19, *P* = 0.05), but not to CSF MHPG, as the reduced model for Alzheimer’s disease-type dementia CSF MHPG only included the volume of CSF, and that older age was related to higher CSF NA (*t* = 2.40, *P* = 0.03) but not to CSF MHPG in the Parkinson’s disease group (*t* = 1.44, *P* = 0.16) (See Table 1). Due to the amount of missing data exceeding the threshold set for inclusion, years post diagnosis (‘ypd’) could not be evaluated in any regression, and disease severity could only be evaluated in Alzheimer’s disease-type dementia NA, where it did not predict CSF NA levels significantly.

**Table 1:** Stepwise regression analyses on ‘dataset 1’—coefficients in the reduced models

Group	Reduced Model	Stat. Sign. Coeff.	Est.	Std.Err	*t*-value	Pr(>|*t*|)
ADD_MHPG	~csfvol	Intercept	10.149	1.455	6.976	0.000***
		csfvol	2.285	1.485	1.539	0.137
ADD_NA	~*n* + age + severity	Intercept	266.80	31.35	8.509	0.000***
		*n*	70.90	32.38	2.189	0.049*
		age	55.73	32.38	1.721	0.1109
		severity	−45.96	32.38	−1.419	0.1813
PD_MHPG	~*n* + age	Intercept	11.243	1.999	5.625	0.000***
		*n*	3.910	2.329	1.679	0.106
		age	3.372	2.329	1.448	0.160
PD_NA	~method + age	Intercept	158.06	35.88	4.406	0.000***
		method RM	128.56	65.95	1.949	0.071
		method LC-ED	159.15	90.48	1.759	0.100
		age	71.34	29.65	2.406	0.030*
CONTR_MHPG	~*n*	Intercept	10.115	1.199	8.435	0.000***
		*n*	1.784	1.227	1.453	0.162
MHPG	~*n* + csfvol + age	Intercept	10.5475	0.9570	11.021	0.000***
		*n*	2.3455	0.9946	2.358	0.021*
		csfvol	1.4250	0.9829	1.450	0.1515
		age	2.1197	1.0007	2.118	0.038*
NA	~*n* + age	Intercept	269.98	36.39	7.418	0.000***
		*n*	67.70	38.25	1.770	0.083
		age	64.57	38.25	1.688	0.098

The variables *n* and *age* significantly predict CSF NA (*t* = 2.19, *P* = 0.05; *t* = 2.40, *P* = 0.03) in the Alzheimer’s disease and Parkinson’s disease group, respectively. Both variables were significant predictors of MHPG (*t* = 2.36, *P* = 0.02; *t* = 2.12, *P* = 0.04) across groups. ADD = Alzheimer’s disease dementia; Coeff = coefficient; ED = electrochemical detection; Est = parameter estimates; LC = liquid chromatography; MHPG = 3-methoxy-4-ydroxyphenylglycol; *n* = Sample size; NA = noradrenaline; PD = Parkinson’s Disease; Pr(>|*t*|) = *P*-value associated with the *t* statistic; RM = radioenzymatic methods; RP = reversed-phase; Stat.Sign.Coeff = statistically significant coefficient; Std.Err = standard error; UHPLC = ultra high performance liquid chromatography. Significance levels are indicated by asterisks (*P < 0.05, ***P < 0.001).

Among the data collapsed across groups and divided only with respect to the noradrenergic measures (NA and MHPG), sample size and age were positively related to CSF MHPG levels (*t* = 2.36, *P* = 0.02; *t* = 2.12, *P* = 0.04) while no such effect was observed for CSF NA (*t* = 1.77, *P* = 0.08; *t* = 1.69, *P* = 0.10).

Given the significant influence of age on NA levels in Parkinson’s disease, a further regression model (Supplementary Table 4, section ~*n* + ‘method’ + ‘csfvol’ + ‘age’*‘severity’ + ‘age’*‘ypd ’) was performed on both NA and MHPG levels to explore the interaction between age and thevariables ‘ severity’ and ‘years post diagnosis’, previously excluded due to their absence for more than half of the participants. For both variables, NA and MHPG, the interaction terms ‘age*severity’ and ‘age*ypd’ were added to the full models. No collinearity between severity, years post diagnosis and age was found for either NA or MHPG CSF levels. The models were not significant (Supplementary Table 4, *P* = 0.21, *P* = 0.48) and did not explain more variance compared to the full models without the interactions, as revealed by the ANOVA results (*P* = 0.29; *P* = 0.90, Supplementary Table 7). None of the variables in the models (*n*, ‘method LC-ED’ , ‘method MF, ‘method GC’ , ‘method GLC’, ‘method RM’, ‘csfvol’, ‘age’, ‘severity’, ‘ypd’, ‘age*severity’, ‘age*ypd’) were significant (Table 2).

**Table 2:** Regression analyses on dataset 1 with additional interaction terms coefficients

Group	Stat. Sign. Coeff.	Est.	Std.Err	*t*-value	Pr(>|*t*|)
PD_MHPG	Intercept	12.333	2.892	4.264	0.000594***
	*n*	2.611	3.587	0.728	0.477173
	method LC-ED	3.737	11.341	0.329	0.746052
	method MF	−44.624	51.843	−0.861	0.402089
	method GC	−3.461	26.520	−0.130	0.897799
	method GLC	−23.880	16.270	−1.468	0.161562
	csfvol	3.736	3.522	1.060	0.304664
	age	5.870	4.270	1.375	0.188225
	severity	5.933	9.209	0.644	0.528502
	ypd	4.228	8.358	0.506	0.619799
	age* severity	9.412	16.634	0.566	0.579335
	age* ypd	5.020	7.299	0.688	0.501489
PD_NA	Intercept	128.713	54.928	2.343	0.0472*
	*n*	−8.559	39.928	−0.214	0.8356
	method LC-ED	205.982	127.914	1.610	0.1460
	method RM	185.784	114.636	1.621	0.1438
	csfvol	30.076	59.731	0.504	0.6282
	age	90.557	87.155	1.039	0.3292
	severity	38.585	47.864	0.806	0.4435
	ypd	−7.809	40.281	−0.194	0.8511
	age*severity	−198.496	133.884	−1.483	0.1765
	age*ypd	−2.316	104.947	−0.022	0.9829

A further regression was performed on both NA and MHPG levels to explore the interaction between the variable ‘age’ and the variables ‘severity’ and ‘years post diagnosis’, previously excluded due to their absence for more than half of the participants. For both variables, NA and MHPG, the tested regression model was then *n* + ‘method’ + ‘csfvol’ + ‘age’*‘severity’ + ‘age’*‘ypd’. Coeff = coefficient; ED = electrochemical detection; Est = parameter estimates; GC = gas chromatography; GLC = gas liquid chromatography; LC = liquid chromatography; MF = mass fragmentography; MHPG = 3-methoxy-4-ydroxyphenylglycol; *n* = sample size; NA = noradrenaline; PD = Parkinson’s Disease; Pr(>|*t*|) = *P*-value associated with the *t* statistic; RM = radioenzymatic methods; Stat.Sign.Coeff = statistically significant coefficient; Std.Err = standard error. Significance levels are indicated by asterisks (*P < 0.05, ***P < 0.001).

## Discussion

Our meta-analysis set out to quantify alterations to the noradrenergic system in Alzheimer’s disease-type dementia and Parkinson’s disease using CSF and PET measures of NA, NA metabolites and NA transporter levels. Effect sizes of the studies included in the meta-analyses (‘dataset 2’) were calculated and pooled. Additionally, exploratory stepwise regression analyses were conducted on ‘dataset 1’ (averages) to investigate associations between CSF NA/MHPG measures and study-related confounds (sample size, analytical method used to evaluate the noradrenergic levels of CSF and volume of the CSF sample) or variables assumed to influence levels of NA and MHPG (age, years post diagnosis and disease severity). We will interpret the results in light of the current literature and discuss the methodological limitations that should be considered when interpreting the results obtained.

In the Parkinson’s disease groups, our observation of a general decrease in noradrenergic measures is consistent with previous literature^4,51,52^ and with post-mortem studies reporting a-synuclein containing Lewy bodies that affect NA synthesis^16^ and/or neuronal cell loss in the LC.^9,15,17,18^ General noradrenergic dysregulation is also implicated in the occurrence of non-motor symptoms in Parkinson’s disease,^20^ such as sleep disorders and autonomic dysfunction that can occur prior to the onset of motor symptoms and become more predominant as the disease progresses.^21^ The results on PET MeNER data show reduced binding in the hypothalamus in Parkinson’s disease, and although binding was reduced also in the LC, median raphe, nucleus ruber and thalamus, these effects were not significant. This was not entirely expected, as we anticipated that the LC and raphe would also be significantly affected considering previous post-mortem studies reporting pathology and cell loss in these structures.^15,18,53–56^ However, in the study of Sommerauer *et al.*,^57^ which was also included in the PET meta-analysis, a significant reduction in NATs density levels were observed in individuals with Parkinson’s disease and with rapid eye movement sleep behaviour disorder (RBD) compared to individuals with Parkinson’s disease alone, in both the LC and raphe. We did not include Parkinson’s disease RBD positive individuals in our meta-analysis to reduce sample heterogeneity with the other study included, thus it will be interesting in future studies to explore to what extent the noradrenergic system is more severely affected in Parkinson’s disease individuals with RBD compared to Parkinson’s disease alone.^58^ Finally, it is conceivable that the limited resolution of PET studies renders it difficult to reliably detect differences in small brainstem nuclei such as the LC and raphe nucleus. Overall, our results in Parkinson’s disease demonstrating decreased CSF NA and MHPG levels compared to their control groups are consistent with the increased degeneration of the noradrenergic system.^20,21^

To explore in more detail how the noradrenergic system may be differentially related to motor and cognitive symptoms in Parkinson’s disease, future studies should assess how CSF measures of NA and MHPG compare between individuals with Parkinson’s disease and Parkinson’s disease dementia.

In the Alzheimer’s disease-type dementia group, we observed increased CSF MHPG levels compared to controls, while no differences were found for CSF NA levels. Measures of MHPG levels obtained directly in the brain tissue of individuals with Alzheimer’s disease show heterogeneous findings, with either increased, decreased^33,59,60^ or unchanged^32^ MHPG levels compared to controls. However, evidence from well-controlled animal studies suggest that differences to the noradrenergic system observed in tissue may be disconnected from those observed in extracellular levels,^34–36,61,62^ and therefore also from CSF levels, which may explain why some studies have found conflicting results. However, the results reported here are consistent with a number of CSF studies (Supplementary Table 1) that we could not include in the meta-analysis (‘dataset 1’) as they did not provide data in a format suitable for calculating effect sizes. Moreover, CSF MHPG, more than NA, seems to be linked to Alzheimer’s disease brain pathology measures i.e. phospho-tau, as animal studies have shown that a NA O-methylation to MHPG is necessary for tau spreading.^63^ In the presence of amyloid and tau biomarkers, CSF MHPG levels were found to improve diagnostic accuracy between Dementia with Lewy bodies/Parkinson’s disease dementia and Alzheimer’s disease^41^ and to be significantly linked to Alzheimer’s disease memory deficits,^40^ suggesting CSF MHPG as a more sensitive measure than CSF NA in the context of differential diagnosis of Alzheimer’s disease and symptom characterization. While increased levels of CSF MHPG thus appear to emerge as a consistent phenomenon in Alzheimer’s disease-type dementia, their occurrence is as of yet not completely understood. It can be speculated that the elevated CSF MHPG levels in the absence of significant changes of CSF NA compared to controls could be due to a desynchronization between the amount of noradrenergic production (NA) and breakdown (MHPG). Coupled with the concurrent dysfunction of adrenergic receptors implicated in cognitive functions,^36^ this might lead to the exacerbation of the cognitive symptoms of Alzheimer’s disease, and therefore be a more distinctive indicator than CSF NA for Alzheimer’s disease-type dementia-related disease and disease progression.

It would be of interest in future studies to investigate differences in MHPG and NA levels in participants with mild cognitive impairment, to determine if noradrenergic dysfunction occurs early or during the transition between mild cognitive impairment and Alzheimer’s disease.

The results of the exploratory regression analyses on ‘dataset 1’ show that NA measures were influenced by the sample size (*n*) in the Alzheimer’s disease-type dementia group, with larger sample sizes being linked to higher CSF NA levels in Alzheimer’s disease. At present it is difficult to interpret this finding as we have currently not enough data to link sample sizes with disease severity or years post diagnosis, which would be expected to influence CSF NA levels and likely vary systematically with access to larger clinical cohorts. It suggests, however, that future meta-analyses should aim to explore interindividual variability in CSF NA measures in conjunction with differences across cohorts.

Furthermore, in the Parkinson’s disease group, increased age was related to higher CSF NA levels. Correlations between noradrenergic levels and age in the literature report mixed results, but considering the large-study sample size (PD NA *n* = 132; PD MHPG *n* = 257) of this exploratory analysis, these results can be considered a reliable indication of a particular correlation between age and CSF NA present in Parkinson’s disease. A larger sample size and age were also found to be linked to higher CSF MHPG levels across groups suggesting that the influence of these variables might be true for MHPG levels across groups but were not observed within each group separately. Without sufficient data on potential mediators of this link, such as years post diagnosis and disease severity, these results are difficult to interpret but suggest the importance of exploring interindividual differences in CSF indicators of NA as well as their dependence on disease progression.

The absence of a relationship between the analytical method and the volume of CSF in the sample with both noradrenergic measures can be interpreted positively, as it could suggest the absence of significant differences between the protocols used by the laboratories, and thus good comparability of CSF data reported in these studies.

### Study limitations

Studies using identical datasets were removed when duplicate samples were reported, however the origin of samples from 41 studies was not reported so the removal of all duplicate data cannot be fully ruled out. Only studies whose mean and standard deviation are provided or calculable from other descriptive measures were included in the study, and not all studies reported this data necessary to calculate effect sizes (*k* = 16). Additionally, in the Parkinson’s disease group, disease severity was typically reported using the H&Y scale, however the Unified Parkinson Disease Rating Scale (UPDRS) or the Movement Disorder Society-Sponsored Revision of the Unified Parkinson’s Disease Rating Scale (MDS-UPDRS) would have been more desirable measures to assess associations with motor and non-motor symptom severity.

Meta-analyses invariable have to contend with unknown relevant aspects of the study samples. Among the included studies reporting NA levels in Alzheimer’s disease, control subjects in four studies were reported to have other comorbidities.^64–67^ Moreover, despite neurological and psychiatric problems being ruled out, other diseases for which controls were hospitalized (*k* = 4) might have influenced noradrenergic levels. The stress ^68^ caused by hospitalization of individuals with Alzheimer’s disease-type dementia and Parkinson’s disease may have also influenced the results reported in our meta-analysis. Also, the majority of studies did not confirm absence of pathology in the control group, thus the presence of preclinical Alzheimer’s disease cannot be ruled out. Similarly, in studies that reported medication status, participants were split into separate subgroups, however this information was not available for all studies. Whilst the majority of studies reported no difference between medicated versus unmedicated participants, we cannot entirely rule out an effect of medication on group differences in NA/MHPG.

There are also still open questions regarding the comparability of MHPG and NA as biomarkers of noradrenergic function. In contrast to NA, MHPG rapidly diffuses through the blood–brain barrier^69^ and blood–CSF barrier.^70^ Thus CSF MHPG levels might not directly correlate with central noradrenergic metabolism.^69^ In this respect, we should be prudent about indicating it as a pure index of central noradrenergic function and interpreting results as such. In this regard, it is also interesting to investigate whether the discrepancies we observed in MHPG levels between Alzheimer’s disease-type dementia and Parkinson’s disease clinical groups (higher in Alzheimer’s disease-type dementia, lower in Parkinson’s disease) might in part be related to peripheral MHPG differences between those clinical groups.

Furthermore, in order to facilitate the use of noradrenergic biomarkers in the future, it will be important to understand the relationship between levels in the CSF and blood more thoroughly. Knowing whether and with which protocols blood noradrenergic measures can be expected to approximate the levels in CSF, and to what degree they relate to noradrenergic dysfunction in the brain, would facilitate the use of such measurements in future studies since blood sampling is a less invasive intervention and more easily tolerated by study participants.

Finally, the definition of the Alzheimer’s disease-type dementia group in the present study is quite broad as it also includes pathologically unconfirmed cases. As easily accessible measures of Alzheimer’s disease pathology in blood/plasma are a fairly recent scientific development (amyloid, phospho-tau and total-tau), most of the articles included in the analyses did not provide pathological confirmation, and exclusion of these would have compromised the completeness of the review and meta-analysis.

In Parkinson’s disease, future studies should aim to more clearly distinguish between idiopathic and atypical Parkinsonian syndromes and seek to understand how CSF and PET biomarkers of noradrenergic dysfunction are related to pathology i.e. via assessment of alpha-synuclein levels in CSF, and if and how those measures correlate with RBD, a potential prodromal marker of Parkinson’s disease that has been previously shown to be related to noradrenergic dysregulation.^71–73^ For future studies in Alzheimer’s disease, the sample characterization should include CSF or blood/plasma measures of phospho-tau, total-tau and amyloid beta ratio 42/40 levels and include cognitive tests that are more closely associated with the noradrenergic system i.e. episodic memory^74,75^ or response inhibition.^76–79^

Future meta-analyses will hopefully be able to summarize a sufficient number of studies with pathology measures, and in order to ascertain to what extent they can explain the differences in NA indicators we have observed in Alzheimer’s disease-type dementia and Parkinson’s disease as compared to healthy controls as well as the heterogeneity in NA indicators observed across individuals with Parkinson’s disease/Alzheimer’s disease-type dementia.

## Conclusion

Determining how the noradrenergic system can be assessed using CSF and PET measures will be beneficial for understanding how changes to this neuromodulatory system contribute to the clinical manifestations of Alzheimer’s disease and Parkinson’s disease. The opportunity to monitor the status of the noradrenergic system using CSF and PET measures may also aid in the early detection of pathological decline and be useful for determining the efficacy of NA drugs in clinical trials.

In this review and meta-analysis, we provided an overview and quantitative assessment of noradrenergic differences reported to date in aging, Alzheimer’s disease-type dementia and Parkinson’s disease assessed in CSF and PET. Overall, these results indicate that CSF measures of noradrenergic dysfunction may be differently altered in both Alzheimer’s disease and Parkinson’s disease. However, further studies are required from pathologically (alpha-synuclein, phospho-tau, total-tau and amyloid) and cognitively characterized cohorts using medication and pathology-free, age-matched control groups to elucidate how these measures correlate with symptom severity and are influenced by Alzheimer’s disease and Parkinson’s disease pathology.

## Supplementary Material

fcad085_Supplementary_Data

## Abbreviations

AD =: Alzheimer’s dementia
ADD =: Alzheimer’s disease dementia
AIC =: Akaike information criterion
CI =: confidence interval
CONTR =: controls
H&Y =: Hoehn and Yahr scale
HKSJ =: Hartung–Knapp–Sidik–Jonkman
LC =: Locus coeruleus
MDS-UPDRS =: movement disorder society-sponsored revision of the unified Parkinson’s disease rating scale
MeNer =: (s, s)-11c-2-(a-(2-methoxyphenoxy)benzyl)morpholine
MMSE =: mini-mental state examination
NATs =: noradrenaline transporters
PD =: Parkinson’s disease
PRISMA =: preferred reporting items for systematic reviews and meta-analyses
RBD =: rapid eye movement sleep behaviour disorder
SD =: standard deviation
SMD =: standardized mean differences
UPDRS =: unified Parkinson’s disease rating scale
