# Supplementary material for: Cerebrospinal fluid and positron-emission tomography biomarkers for noradrenergic dysfunction in neurodegenerative diseases: a systematic review and meta-analysis
Source: Brain Commun. 2023 Mar 29;5(3):fcad085. doi: 10.1093/braincomms/fcad085 (PMC10154713; doi:10.1093/braincomms/fcad085)
Supplement: fcad085_Supplementary_Data [file fcad085_supplementary_data.docx]

**Supplementary Material
CSF and PET biomarkers for noradrenergic dysfunction in neurodegenerative diseases: a systematic review and meta-analysis.**

Elisa Lancini ^1, 2^, Lena Haag ^1, 2^, Franziska Bartl ^2^, Maren Rühling ^2^, Nicholas J. Ashton ^3, 5, 6, 7^, Henrik Zetterberg ^4, 8, 9, 10, 11^, Emrah Düzel ^1, 2, 12, 13^, Dorothea Hämmerer ^1, 2, 12, 13, 14 *^, Matthew J. Betts ^1, 2, 13 *^.

^*^ These authors contributed equally

1. German Center for Neurodegenerative Diseases (DZNE), Otto-von-Guericke University Magdeburg, Magdeburg, Germany.
2. Institute of Cognitive Neurology and Dementia Research (IKND), Otto-von-Guericke University Magdeburg, Magdeburg, Germany.
3. Institute of Psychiatry, Department of Old Age Psychiatry, King’s College London, London, UK.
4. Department of Psychiatry and Neurochemistry, the Sahlgrenska Academy at the University of Gothenburg, Mölndal, Sweden.
5. Wallenberg Centre for Molecular and Translational Medicine, University of Gothenburg, Gothenburg, Sweden.
6. NIHR Biomedical Research Centre for Mental Health & Biomedical Research Unit for Dementia at South London & Maudsley NHS Foundation, London, UK.
7. Department of Psychiatry and Neurochemistry, Institute of Neuroscience & Physiology, the Sahlgrenska Academy at the University of Gothenburg, Mölndal, Sweden.
8. Clinical Neurochemistry Laboratory, Sahlgrenska University Hospital, Mölndal, Sweden.
9. Department of Neurodegenerative Disease, UCL Institute of Neurology, London, UK.
10. UK Dementia Research Institute at UCL, London, UK.
11. Hong Kong Center for Neurodegenerative Diseases, Hong Kong, China.
12. Institute of Cognitive Neuroscience, University College London, London, UK.
13. Center for Behavioral Brain Sciences, University of Magdeburg, Magdeburg, Germany.
14. Department of Psychology, University of Innsbruck, Innsbruck, Austria.

**Corresponding author:**

Elisa Lancini

Institute of Cognitive Neurology and Dementia Research, Leipziger Str. 44, Magdeburg

[elisa.lancini@dzne.de](mailto:elisa.lancini@dzne.de)

**CONTENTS**

Page 3 Supplementary Methods

Page 5 Additional Analyses and results

Page 7 Supplementary Figure 1. Risk bias assessment (*dataset 2*)

Page 8 Supplementary Figure 2. Influence analysis and Graphic Display of Heterogeneity (GOSH) plot analysis (*dataset 2*)

Page 11 Supplementary Figure 3. Factor variables “Method” and “Group” for *dataset 1* - MHPG

Page 12 Supplementary Figure 4. Forest plot of MHPG levels (Means and SD) in CSF

Page 13 Supplementary Figure 5. Forest plot of NA levels (Means and SD) in CSF

Page 14 Supplementary Table 1. Qualitative synthesis

Page 34 Supplementary Table 2. Meta-analysis results (*dataset 2*)

Page 35 Supplementary Table 3. Weighted averages

Page 36 Supplementary Table 4. Regressions on *dataset 1 (fullmodels)*.

Page 38 Supplementary Table 5. Stepwise regressions on *dataset 1*

Page 39 Supplementary Table 6. ANOVA analysis. Comparison between full models and reduced models.

Page 40 Supplementary Table 7. ANOVA analysis. Comparison between full models and full models with additional interaction terms (*age*severity* and *age*ypd*).

Page 41 Supplementary References

## Supplementary Methods

**Keywords:**

We searched PubMed for English relevant articles using the keywords “CSF noradrenaline”,

“PET noradrenaline”, “PET noradrenergic”, “CSF noradrenergic”, “PET MeNER”' together with

“Parkinson”, “Alzheimer”, “dementia”, “ageing”, “aging”, “neurodegeneration”.

**Search strategy:**

Four authors (E.L, M.R, L.H, F.B) reviewed and selected the articles based on the abstracts.

When there was disagreement between the selected studies, the authors reached a consensus via discussion. The enrolled studies were then reviewed for inclusion criteria by E.L. The criteria for inclusion were: (i) the presence of CSF and PET noradrenergic biomarkers measures; (ii) clear sample composition; (iii) clear descriptive statistics of sample size and biomarker measures; (iv) clear description of methodology.

**Selection criteria:**

The literature search in PubMed ended in January 2021 and resulted in 509 articles (CSF= 302; PET= 207). An additional 13 articles were identified in the references of these articles

identified on PubMed. After duplicate removal, the remaining 264 articles (CSF= 141; PET=

123) were screened and a further 26 articles were excluded since they were unavailable

online (CSF= 11; PET= 15). An additional 172 articles (CSF= 69; PET= 103) were excluded as unrelated to the purpose of this review (i.e. animal studies, studies not in ADD or PD or studies not related to the noradrenergic system). From the remaining 66 articles (CSF=61; PET= 5), 16 were excluded because (i) data was missing, (ii) not reported as mean and standard deviation or in a format that did not allow for transformation into mean and standard deviation (iii) or because the reported data was already present in other studies included in the meta-analysis. In the event that the same sample was shared across studies, we included the study that was considered to be most relevant to the scope of the meta-analysis. Data from the remaining 50 studies (CSF=46; PET=4) was collected and any missing data was requested by contacting the authors of articles eligible for the meta-analysis. Finally, 26 studies reported a suitable comparison between healthy controls and ADD/PD and sufficient data (mean and SD) for the calculation of the meta-analysis (CSF=23; PET=3).

Studies in which the control groups were not age-matched to the ADD/PD groups were included to the extent that these studies were not outliers in the analysis and age dissimilarity

was not a reported concern in the original articles. The control groups included are those in which major neurological problems, dementia and cognitive deficits were excluded. Possible biases due to the inclusion of control groups from articles in which the control group included persons with other minor neurological problems are discussed in the “Additional Analyses and results” section.

**Database control:**

The database control revealed that studies from Abdo 2007, Chia 1995, Raskind 1999 and Tohgi 1990 and Mayeux 1984 ^1–5^ used the same database as Abdo 2004, Chia 1993, Peskin 1995, Tohgi 1993 and Mayeux 1986 respectively ^6–10^. The former articles were excluded and the latter retained as they reported either the same number of subjects ^8^, a higher number of subjects ^6,7^, or more information^4,10^ compared to the articles using the same subjects. Freed, 1989 and Mayeux, 1986 used the same control subjects ^10,11^, thus only Freed, 1989 ^11^ is reported in the overall analysis. No ante-mortem studies using PET MeNER in ADD were found.

### **Data transformations**

Measures of NA and MHPG in the CSF and the density of NA transporters (NETs) in the brain were extracted for all studies included in the meta-analysis. To uniform data across studies, both CSF and PET data were first transformed to mean and standard deviation measures. When data was reported using the median and interquartile range but followed symmetrical distribution, the median was used as mean and the standard deviation calculated as interquartile range *(Q3-Q1)/1.35*. Second, additional transformations were applied for CSF and PET data separately. For CSF NA and MHPG measures, data was converted to the standard unit of measure, i.e., picograms or nanograms per milliliter (pg/mL, ng/mL) respectively using GraphPad. For PET studies, data were kept in the unit of measure reported, non-displaceable binding potential (ND-BP) ^12,13^ or distribution volume ratios (DVRs) ^14^, and when measures were reported for left and right hemisphere separately ^13^, the mean and standard deviation of each bilateral region was reported. The tissue model was the reference tissue model 2 (SRTM2) in all included papers. Finally, for studies that used the same control group to compare with different ADD and PD subgroups the number of participants in the control group was split between the subgroups to reduce the unit-of-analysis error ^15^. This step was not necessary for PET data because no studies reported data from both ADD and PD.

## Additional Analyses and results

To assess absolute differences in CSF measures across studies and between groups (CONTR; ADD; PD), data from this second dataset was used to create a forest plot of means and standard deviations (Supplementary Fig. 4, Supplementary Fig. 5). The arithmetical averaged mean was calculated for each group (CONTR; ADD; PD) and measure (MHPG; NA), and a value of 2 standard deviations from the arithmetical averaged mean was used to detect outlier data in every group. Studies identified as outliers were removed from subsequent analyses. An averaged mean and SD were calculated for each group (CONTR; ADD; PD) and measure (MHPG; NA). Differences in the averaged mean between groups were calculated using the Kruskal-Wallis test. This analysis was not conducted for PET studies as the groups (PD; CONTR) were composed of only two studies. For the data visualization plot, weighted averaged means of the groups were calculated using the sample size as weight (Supplementary Table 3).

The analyses did not show any significant difference (NA: X2= 2.23, df= 2, p-value= 0.327; MHPG: X2= 0.96, df= 2, p-value=0.62), thus are not in line with the meta-analysis results that showed increased MHPG levels in ADD and a general noradrenergic decrease in PD. This discrepancy might be due to various factors. As the meta-analysis on *dataset 2* was based on effect sizes in group differences, while the analysis on *dataset 1* was based on absolute group-specific values, more significant results in the difference-based analysis could be due to a positive publishing bias. Also, heterogeneity of samples and methods should be more pronounced in datasets that did not assess differences between controls and clinical groups. Heterogeneity in results between studies may be due to several reasons such as different inclusion criteria, for example the inclusion of "age-matched" control subjects was not always considered; the inclusion of controls who were considered healthy, but might suffer from other diseases (Martignoni et al., 1991, 1992: hospitalized for diagnostic purposes, Stefani et al., 2015: radiculopathies, Tohgi et al., 1992: tension headache) ^16–19^, in which an influence on the noradrenergic system could not be totally ruled out. For example, in the study by Tohgi and colleagues ^19^, tension headache was not considered an exclusion criteria for healthy participants. However a more recent study hypothesized an association between tension headache and dysfunctional noradrenergic pain modulation ^20^. Thus, in this case, tension headache might have introduced a bias in the comparison between healthy and clinical groups. Moreover, the absence of pathological confirmation makes this point one of the most likely sources of bias for the standardization of the samples across studies. Finally, differences in sensitivity and specificity of CSF analytical techniques used between studies and subsequent development of these techniques over the years may have influenced comparisons across studies. Differences in CSF noradrenergic levels within studies may also be present and affect the comparability of results even within the study itself, as other medications besides levodopa (ADD: Palmer et al., 1984; PD: Turkka et al., 1987, AD and PD: Janssens et al., 2018) ^21–23^, were also discussed by Janssens et al., (2018) ^23^ to be a possible cause of bias in their results. Finally, our comparison of group measures summarized across studies (*dataset 1*) might also have been methodologically more prone to be influenced by heterogeneity in sample sizes, as it is not possible to calculate weighted means accounting for sample sizes in the Kruskal-Wallis test. Of course, difference-based and absolute value-based meta-analyses should ultimately yield the same results.

**Supplementary** **Figure 1. Risk bias assessment (*dataset 2*).** Four authors (E.L, M.R, L.H, F.B) individually assessed the quality of every article (“high”, “low”, and “unclear”) under five different domains. The four rating tables were then merged and uploaded into the online *robvis* visualization tool. The output is a plot where the domains-related and overall judgements are expressed with a “traffic light” system. D1: bias arising from the randomization process (pathology characterization); D2: bias due to deviations from intended interventions; D3: bias due to missing outcome data (data reported in the paper or extracted from plots); D4: bias in the measurement of the outcome (information on CSF analysis method or PET tracer used); D5: bias in the selection of the reported result.


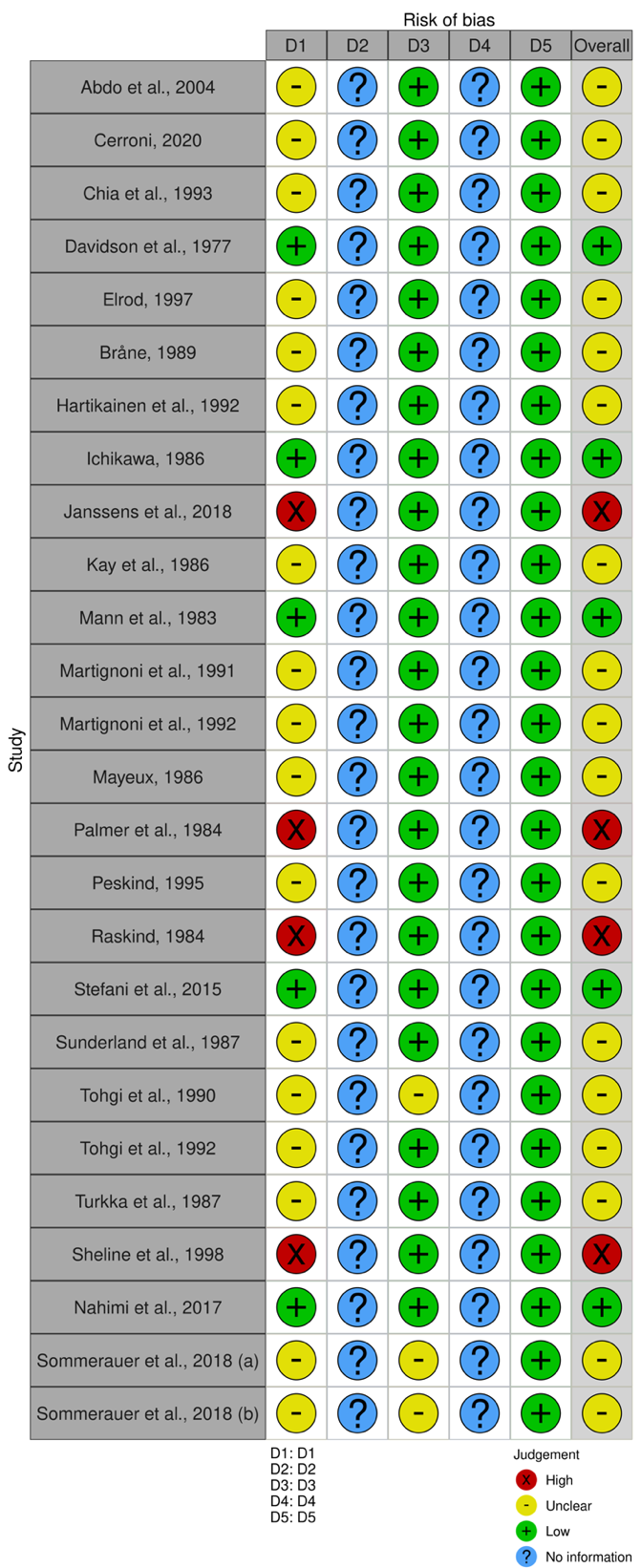


**Supplementary** **Figure 2. Influence analysis and Graphic Display of Heterogeneity (GOSH) plot analysis (*dataset 2*).** The figure describes the process used to detect the patterns of effect sizes and heterogeneity in the data after classical outlier removal based on standard deviations. (**a**) Influence analysis with *InfluenceAnalysis* R function. The function is based on the Leave-One-Out method and recalculates the results $K-1$ times, each time leaving out one study. It assesses possible distortions in the pooled effects by detecting studies which influence the overall estimate the most. The output are plots showing the differences with and without the inclusion of each study. Here we reported the DIFFITS value and the Cook’s distance plots. These measures indicate respectively (i) how much, in terms of standard deviations, the predicted pooled effect changes after excluding this study and (ii) the distance between the values once the study is included or excluded. Values in red were identified automatically as outliers using the cut off proposed by Viechtbauer & Cheung ^24^. (**b**) The Graphic Display of Heterogeneity ^25^ fits the meta-analysis model to all possible subsets ($2^^{k-1}$ possible study combinations). The results are a plot displaying the pooled effect size on the x-axis and the between-study heterogeneity at the y-axis, allowing us to look for specific subclusters in our data. Symmetrical distributions indicate that the effect sizes in our sample are homogeneous, asymmetrical distributions and peaks indicate the presence of subclusters (e.g in the NA PD group two peaks indicate two subclusters, that are found to be driven by study n°1 and n°6). The between-study heterogeneity (I-squared) is the percentage of variability in the effect sizes, not caused by sampling error. (**c**) Using the *gosh.diagnostics* function, three clustering algorithms (k-means, DBSCAN, Gaussian Mixture Model) detect clusters in the GOSH plot data and which studies contribute to create them. The studies reported are subsequently excluded from analyses. The turquoise points represent the distribution of the results when the selected study is included.


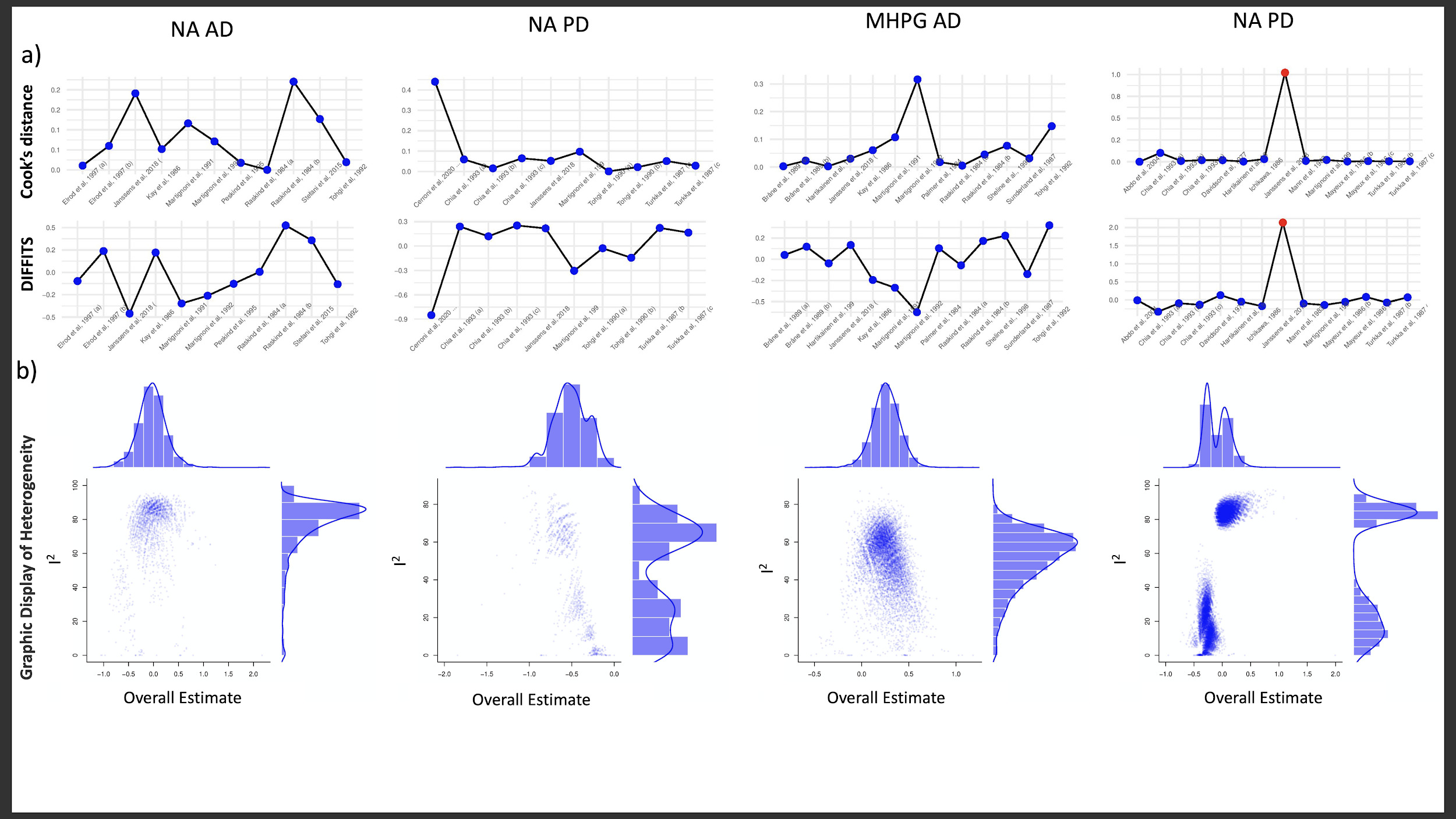


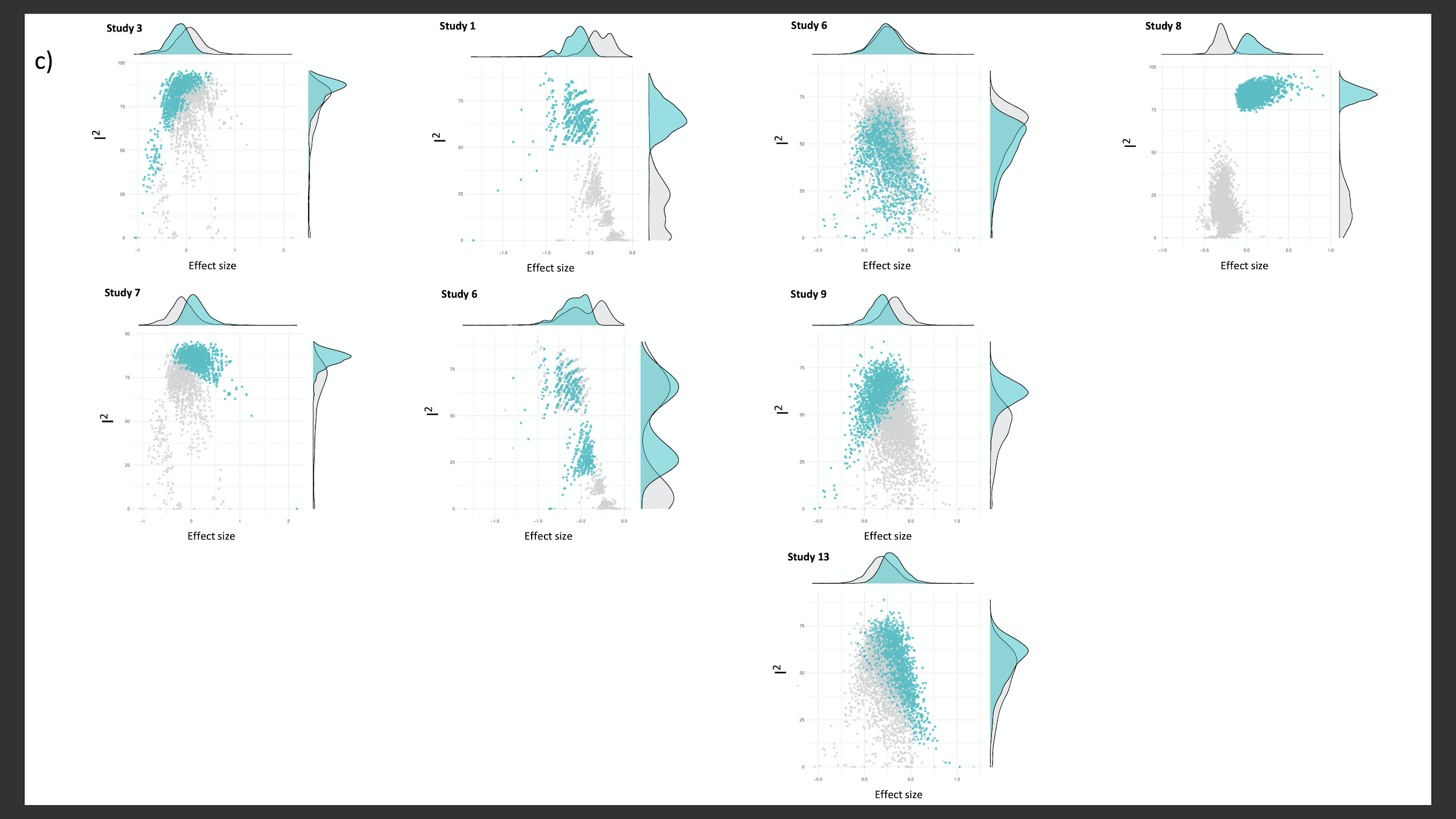


**Supplementary** **Figure 3. Factor variables “Method” and “Group” for *dataset 1* - MHPG.** The figure shows the functions used to convert the variables “Method” and “Groups” into factors and create the relative regressors. The steps reported in the figure are shared across all analyzed groups (MHPG AD; MHPG PG; MHPG CONTR; NA AD; NA PD; NA CONTR; NA; MHPG) while the output differs for every analyzed group, as not all groups had the same number of levels (*K*) in the “Method” variable. In this figure, we report the results for the MHPG group.

In all groups, the variables are first (#1) converted to factors using the function *as.factor()*. Then (#2), the reference level of each factor (“HPLC” for the variable “Method”, “CONTR” for the variable “Groups”) was specified using *relevel()* function. Finally (#3), we explored the regressors obtained from the reference specification, using *contrasts()* function. This function returns a contrasts matrix with *K* rows and *K*-1 columns. The rows report the levels of the factor variable (*K*), with the reference value in the first row. The columns represent the regressor calculated for each factor level based on the reference value assigned.

HPLC = high performing liquid chromatography; GC/MS = gas chromatography/mass spectrometry; LC-ED = liquid chromatography electrochemical detection; MF = mass fragmentography; GC = gas chromatography ; GLC = gas liquid chromatography.

**
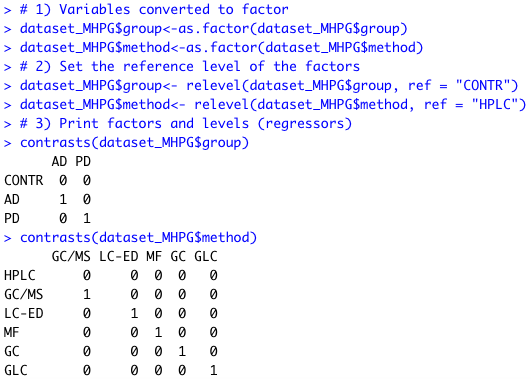
**

**Supplementary** **Figure 4. Forest plot of MHPG levels (Means and SD) in CSF.** Forest plot of means and standard deviations data originated from all articles reporting data points, regardless of their inclusion or exclusion in the meta-analysis. For each study, all datapoints reporting CSF NA or MHPG levels are included. Weighted averaged means of the groups were calculated using the single studies sample sizes as weight. The averages of the weighted means are indicated by the diamonds. CSF = cerebrospinal fluid; MHPG = 3-methoxy-4-hydroxyphenylglycol; ng/mL = nanograms per milliliter.


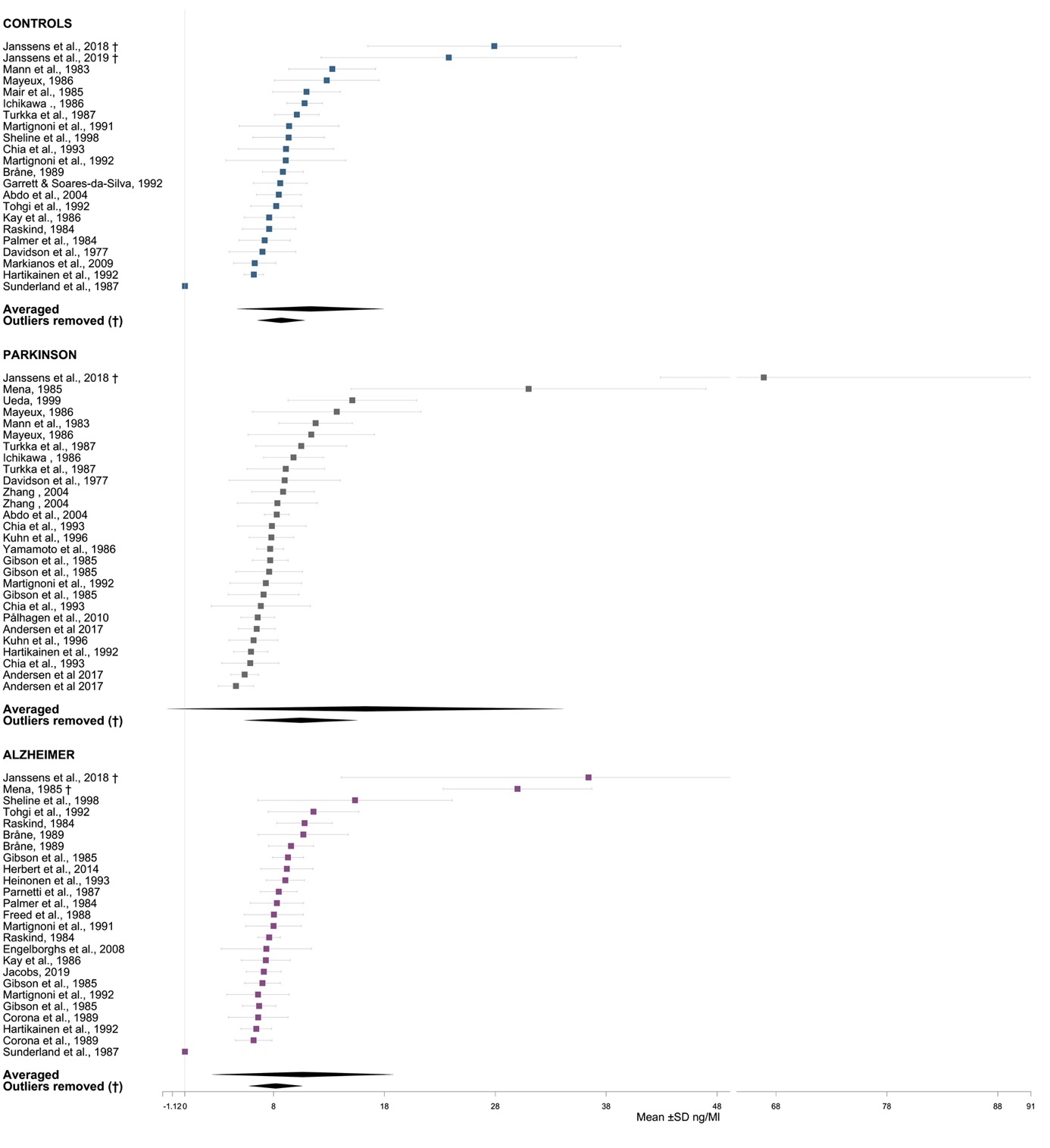


**Supplementary** **Figure 5. Forest plot of NA levels (Means and SD) in CSF (*dataset 1).*** Forest plot of means and standard deviations data originated from all articles reporting data points, regardless of their inclusion or exclusion in the meta-analysis. For each study, all datapoints reporting CSF NA or MHPG levels are included. Weighted averaged means of the groups were calculated using the single studies sample sizes as weight. The averages of the weighted means are indicated by the diamonds. CSF = cerebrospinal fluid; NA = noradrenaline; pg/mL: picograms per milliliter.


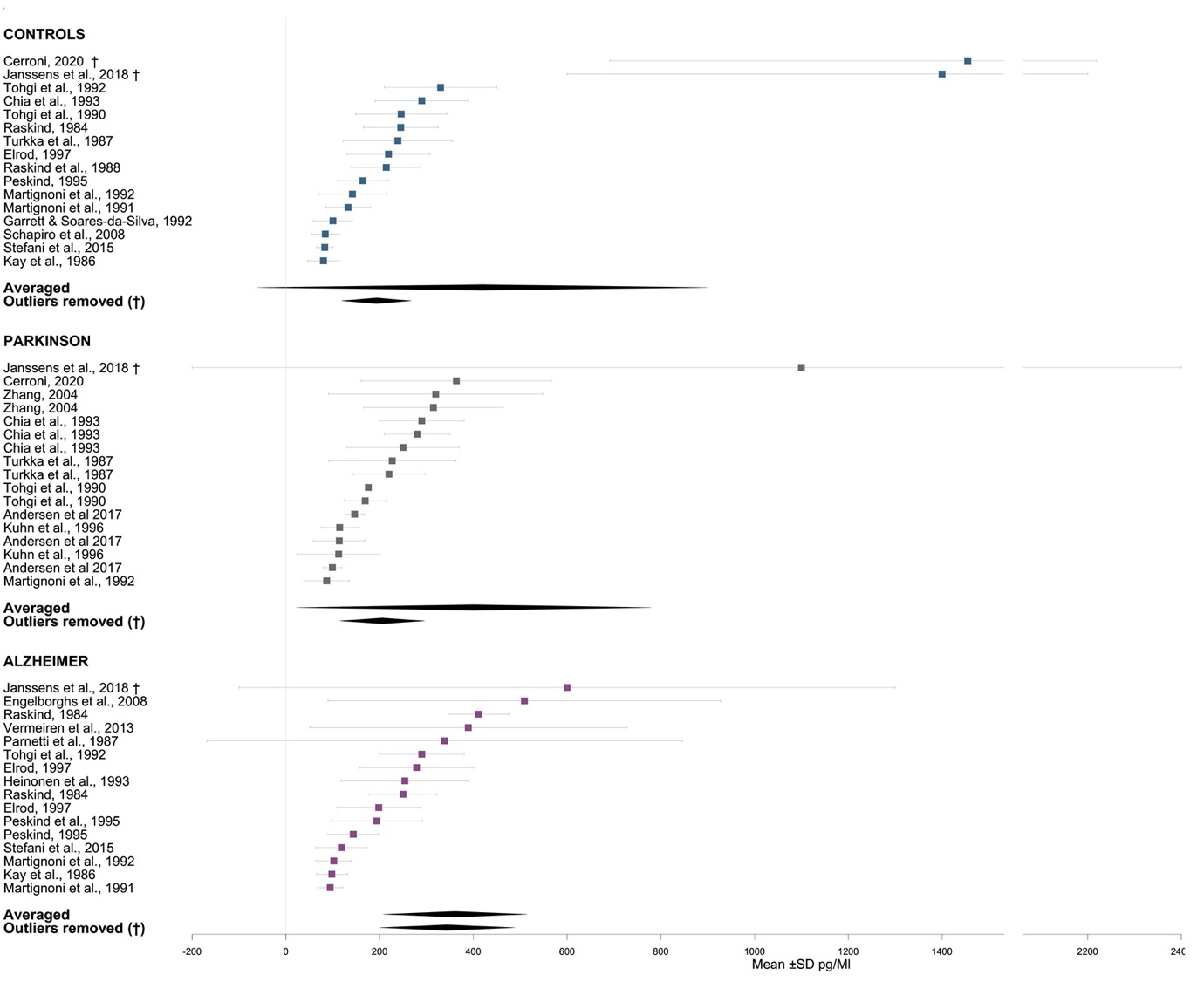


**Supplementary** **Table 1. Qualitative synthesis.** Summary of CSF (k =46) and PET (k =4) studies included in *dataset 1 (*k*= 50)*, of which k =23 (CSF) and k = 3 (PET) included also in *dataset 2 (*k= 26). For each article, we reported only the findings relevant to the purpose of this review and meta-analysis. The clinical groups are reported with the same nomenclature as given in in the articles. 5HIAA = 5-Hydroxyindoleacetic acid; Ab42 = amyloid beta 41; AD = Alzheimer’s dementia; ANS = autonomic nervous system; ANSDS = autonomic nervous system disability scale; BPnd = binding potential non-displaceable; CDR = clinical dementia rating; CONTR = controls; CSF = cerebrospinal fluid; CT = computed tomography; CURS = Columbia university rating scale; DAT = dementia of the alzheimer type; DOPAC = 3,4-Dihydroxyphenylacetic acid; DR = dorsal raphe; DSM = diagnostic and statistical manual of mental disorders; DVRs = distribution volume ratios; ED = electrochemical detection; EMG = electromyography; GBS = Gottfried-Brane-Steen scale; GC = gas chromatography; GDetS = global deterioration scale; GLC = gas liquid chromatography; GMS = Guild memory scale; H&Y = Hoehn and Yahr scale; HDRS = Hamilton depression rating scale; HDS = Hasegawa’s dementia scale; HPLC = high-performance liquid chromatography; L-DOPA = levodopa; L-Threo-DOPS = L-threo-dihydroxyphenylserine; LC = liquid chromatography; LP = lumbar puncture; M = means; MADRS = montgomery-asberg depression rating scale; MDS-UPDRS = movement disorder society-sponsored revision of the unified parkinson’s disease rating scale; MDS = movement disorder society; MeNER = (S,S)-11C-2-(a-(2-methoxyphenoxy)benzyl)morpholine; MF = mass fragmentographic method; MHPG = 3-methoxy-4-hydroxyphenylglycol; MMSE = mini-mental state examination; MOCA = montreal cognitive assessment; MR = median raphe; MRI = magnetic resonance imaging; MS = mass spectrometry; MSQ = mental status questionnaire; n.i = not included in the study; n.s = not specified; NA = noradrenaline; NINCDS-ADRDA = national institute of neurological and communicative disorders and stroke-alzheimer’s disease and related disorders association; NMS = neuroleptic malignant syndrome; p-tau = phospo-tau; PD-L = individuals with Parkinson’s disease receiving L-DOPA, non-dyskinetic; PD-LID = individuals with Parkinson’s disease receiving L-DOPA, dyskinetic; PD = Parkinson’s disease; RBD = rapid eye movement sleep behavior disorder; Ref = reference; RM: radioenzymatic methods; RP = reversed phase; SDAT = senile dementia of the alzheimer type; SPECT = single-photon emission computed tomography; t-tau = total-tau; TR = text revision; UHPLC = ultra-high-performance liquid chromatography; UoM = unit of measure; UPDRS = unified parkinson’s disease rating scale

| **Ref.** | **Controls** | | | **Clinical group** | | | | | | **Method** | **Findings** | **Considerations**  **&**  **Limitations** | **Dataset** |
| --- | --- | --- | --- | --- | --- | --- | --- | --- | --- | --- | --- | --- | --- |
|  | **Measure** | **n** | **Age**  **(M)** | **n** | **Age**  **(M)** | **Clinical Group** | **Severity**  **Based on** | **Treatment** | **Pathology assessment** |  |  |  | ­­­­­­­ |
| ^6^ | MHPG | 23 | 55 | 35 | 53 | PD | H&Y | Withdrawal | UK Parkinson’s  Disease Society Brain Bank criteria. | HPLC | - No differences in CSF MHPG levels between clinical group and controls | 10 individuals in the PD group had parkin gene mutation. | 1,2 |
| ^26^ | NA | 16 | 62 | 14 | 67 | PD | H&Y | Withdrawal | UK Brain Bank  Criteria; beta-CIT; striatal SPECT scanning; excellent motor responses to L-DOPA. | HPLC ED | CSF NA levels were significantly lower in individuals with PD vs controls. |  | 1,2 |
| ^7^ | MHPG  NA | 25  26 | 62  62 | 15  18  8 | n.s for subgroups | PD mild  PD moderate  PD severe | H&Y | Untreated + Withdrawal | H&Y | RP-HPLC-ED | - No significant differences in CSF analytes levels between clinical groups and controls;  - No relation between CSF MHPG or NA levels and severity of PD. | Controls with lower back pain; sciatica; benign prostate hypertrophy; In the MHPG group there are 8 individuals with severe PD, but in the NA group there are 9 individuals with severe PD. | 1,2 |
| ^27^ | MHPG | 5 | 61 | 56 | 62 | PD | Clinical Evaluation | Withdrawal. Fifteen individuals with PD maintained on anticholinergic drugs. | n.s | GLC | - Not significant difference in CSF MHPG levels in individuals with PD compared to controls;  -No difference IN CSF MHPG levels between individuals receiving anticholinergics in the PD group. No effects of L-DOPA;  -There was no correlation between CSF MHPG levels and severity. | Controls with migraine, tension headache, diabetic neuropathy, ocular myopathy, or lumbar disc disease. | 1,2 |
| ^28^ | NA | 42 | 68 | 49  25 | 69  69 | AD mild  AD advanced | MMSE | Withdrawal | NINCDS-ADRDA; DSM IV | Single isotope radioenzymatic method | - Higher CSF NA levels in individuals with advanced AD compared to all other groups;  - Lower CSF NA levels in individuals with mild AD compared to controls;  - Advanced age per se affected CSF NA concentration;  - There were no effects of gender. | Occasional nonprescription  analgesics or laxatives to the clinical groups. | 1,2 |
| ^29^ | MHPG | 26 | 65 | 13  28 | 65  79 | AD  SDAT | GBS; MMSE | Withdrawal | DSM III | GC/MS | - Higher MHPG levels in SDAT clinical group compared to controls.  - Positive correlation between CSF MHPG and restless legs in the AD clinical group. | Controls and individuals with AD are significantly younger than individuals with SDAT | 1,2 |
| ^30^ | MHPG | 34 | 49 | 33  35 | 72  64 | AD  PD | NINCDS-ADRDA; H&Y | Withdrawal | AD: NINCDS-ADRDA; Hachinski’s Ischemic Score; CDR;  Webster’s scale  PD: H&Y | HPLC ED | - No significant differences in CSF MHPG levels between clinical groups and controls;  -Positive correlation of CSF MHPG levels with age in AD clinical group. |  | 1,2 |
| ^31^ | MHPG | 30 | 45 | 11 | 59 | PD * | n.s | Untreated | n.s | HPLC | -No differences in analytes levels between individuals with Parkinsonism and individuals with PD;  - No significant CSF MHPG levels differences between males and females in controls;  - No significant correlation between CSF MHPG levels and age in controls. | *Distinction between Parkinsonism and Parkinson, no details about criteria. | 1,2 |
| ^32^ | MHPG  NA | 43 | 73.8 | 53  52 | 76.6  75.5 | DLB/PDD  AD | n.s | Treated | NINCDS/ ADRDA; DSM IV;  Brain autopsy | RP-HPLC-ED | - Significant increase of diagnostic accuracy between DLB/Parkinson’s disease dementia and AD when MHPG CSF and serum levels were added to the CSF AD biomarker panel;  - Significantly lower CSF NA levels in AD clinical group compared to controls;  - CSF and serum NA levels were significantly higher in individuals free of anti-Parkinson’s medication compared to individuals on medication. | Controls with low back pain, peripheral nervous system disorders, and subjective complaints. Possible presence of non-AD dementia types, which might also display disturbances in monoamine content, in the ‘control’ group. | 1,2 |
| ^33^ | MHPG  NA | 14 | 65 | 30 | 67 | AD | n.s | Withdrawal | DSM II | HPLC | -No significant differences in CSF NA and MHPG levels between clinical group and controls. |  | 1,2 |
| ^34^ | MHPG | 8 | 54 | 17 | 61 | PD | New York University scale, H&Y, MSQ, GMS | Unmedicated + withdrawal | n.s | GC | No difference in CSF MHPG levels compared to controls. | Controls were being investigated for low back pain and headache, but no neurological cause was found. | 1,2 |
| ^16^ | MHPG  NA | 14 | 67 | 14 | 71 | DAT | GDetS | Untreated | NINCDS-ADRDA | RP-HPLC ion pairing | - Lower CSF NA levels in DAT clinical group compared to controls;  -CSF NA levels were significantly inversely correlated with duration of illness;  -CSF NA shows a trend (p = 0.06) toward a positive correlation with MMS. | Controls underwent lumbar puncture for diagnostic purposes. None of them had evidence of focal neurological signs or lumbar disc lesions, and psychiatric disease were ruled out. | 1,2 |
| ^17^ | MHPG  NA | 36 | 61 | 29  22 | 59  64 | PD  DAT | H&Y; CURS | Untreated | NINCDS-ADRDA | RP-HPLC ion pairing | - Lower CSF NA levels in both PD and DAT clinical groups compared to controls;  - No significant correlation of CSF analytes levels with age in PD clinical group. | Controls underwent lumbar puncture for diagnostic purposes. None of them had evidence of focal neurological signs or lumbar disc lesions, and psychiatric disease were ruled out. | 1,2 |
| ^10^ | MHPG | 15 | age-matched | 21  28 | n.s for subgroups | PD (depressed)  PD (not depressed) | Columbia University Parkinson’s  Disease rating scale. | Withdrawal, but some individuals kept assuming anticholinergics | DMS III | HPLC amperometric detection | -No significant differences in CSF MHPG levels between clinical group and controls. | Controls have various neuromuscular disorders but no depression or movement disorder. | 1,2 |
| ^21^ | MHPG | 25 | 60 | 21* | n.s | AD | n.s | Withdrawal | Histologically confirmed | HPLC | -No significant difference in CSF MHPG levels compared to control;  -CSF MHPG levels did not differ between male and females.  -CSF MHPG levels did not correlate with age;  -No differences in MHPG CSF levels due to phenothiazines and/or butyrophenones medications. | *Considered only drug free individuals. | 1,2 |
| ^35^ | NA | 8 | 71.5 | n.i | n.i | AD | MMSE | Withdrawal | NINCDS-ADRDA, DSM III | RM | - In the AD clinical group, CSF NA levels correlated negatively with MMSE and HDS scores;   No significant differences in CSF NA levels when comparing untreated individuals with individuals receiving L-DOPA. |  |  |
| ^36^ | MHPG  NA | 6 | 67 | 7  9 | 61  68 | AD moderate  AD advanced | GDetS | Untreated  Untreated+ withdrawal | DSM III  11 individuals in the AD clinical group were found on post-mortem verification to have had AD. | GC/MS;  RM | - Higher CSF NA levels levels in individuals with moderate AD compared to controls;  - Significantly higher CSF NA and MHPG levels in individuals with advanced AD compared to controls;  - Individuals with advanced AD showed significantly higher levels of both analytes compared to individuals with moderate AD;   - -In the AD clinical group, higher CSF MHPG for individuals with most severe dementia. |  | 1,2 |
| ^18^ | NA | 16 | age-matched | 26 | 70 | AD mild to moderate | Disease duration | n.s | n.s | HPLC | - Higher CSF NA levels in individuals with AD compared to controls. | Controls had dementia ruled out but other neurological diseases (mostly radiculopathies). | 1,2 |
| ^37^ | MHPG | 7 | 62 | 13 | 58 | DAT | CDR | Withdrawal | DSM III | HPLC ED | -No significant differences in CSF MHPG levels between clinical group and controls; - Trend (p = 0.10) for baseline MHPG to decrease with increasing age in the clinical group;  - Trend (p= 0.10) for CSF MHPG levels to increase with increasing severity. |  | 1,2 |
| ^38^ | NA | 31 | 61 | 5  1 | 60  75 | PD | H&Y | Treated  Untreated | H&Y | HPLC ED | - No significant differences in CSF NA levels between clinical group and controls;  - No significant differences of medication between untreated and treated individuals in the PD clinical group. | Controls with minor neurological problems | 1,2 |
| ^19^ | MHPG  NA | 15 | 69 | 11 | 71 | AD/SDAT | HDS | Withdrawal | DSM III-R;  Hachinski’s Ischemic Score; NINCDS-ADRDA; CT; MRI | HPLC ED | - Higher CSF MHPG levels in AD/SDAT clinical groups compared with the controls.  - CSF NA levels correlated negatively with intellectual ability. | Controls with tension headache | 1,2 |
| ^22^ | MHPG;  NA | 14 | 60 | 15  19 | n.s for subgroups | PD | n.s | Treated  Untreated | ANSDS | HPLC ED;  RP-HPLC-ED | - No significant differences in CSF analytes levels between clinical group and controls;  - No effect of medications on CSF analytes levels;  - No correlation between CSF NA levels and the severity of autonomic failure.  - Significant negative correlation between CSF MHPG levels and severity of autonomic dysfunction.  - No influence of the duration of the disease on CSF NA or MHPG levels. |  | 1,2 |
| ^39^ | MHPG | 10 | 75 | 28 | 73 | DAT | CDR; Blessed Scale; Sum of boxes | n.s | ADRC diagnostic criteria; Post-mortem validation | HPLC ED | - Elevated CSF MHPG levels in individuals with DAT vs controls;  - Overall, CSF MHPG levels inversely correlate with cognitive functions. The correlation was not significant within the DAT sample. |  | 1,2 |
| ^40^ | NA,  MHPG | 16 | 55.1 | 7,6,7 | 64.3,67,58.5 | PD, PD-L, PD-LID | ON-stage using the MDS-UPDRS part III, MMSE and MOCA | Untreated, treated with L-DOPA, treated with L-DOPA | Diagnosis made by movement disorder specialists using the UK Brain Bank Criteria for idiopathic PD | HPLC ED | - In PD-L, CSF NA levels were higher as compared to controls and individuals in the PD-N group.  - In PD-LID, CSF NA and MHPG levels were not different from controls.  - In the PD-N group CSF MHPG levels were lower compared to the PD-LID group. | The study included 20 individuals with PD and one Lewy body dementia (DLB), all diagnosed by movement disorder specialists using the UK Brain Bank Criteria for idiopathic PD (Hughes et al. 1992). *Since DLB, like PD, is a synucleinopathy, we included this participant in the PD group.*  Control subjects’ diagnosis: headache n=3, dementia n=3, multiple sclerosis n=3, vertigo n=1, ataxia n=1, trigeminal neuralgia n=1, unspecified sleep disorder n=1, neuroborreliosis n=2. Two controls were diagnosed as having Alzheimer’s disease (AD), One had a possible brain stem or cerebellar disorder, one post concussion syndrome, and one had a potential sleep disorder.  *Therefore, this study was not included in dataset 2 despite reporting a control group.* | 1 |
| ^41^ | MHPG | n.i | n.i | 21 | 65.9 | DAT | CDR | Withdrawal | DSM III, Hachinski score | HPLC ED | - Basal MHPG levels in the CSF were higher in the MID clinical group in comparison with the DAT clinical group;  -No correlations were found between CSF MHPG and age. |  | 1 |
| ^42^ | NA,  MHPG | n.i | n.i | 181 | 79.2 | AD | MMSE, HDS, GDetS | Withdrawal | NINCS/ANRA, DSM-IV, followed up, autopsy | HPLC ED | - CSF NA levels were significantly correlated with DOPAC and 5HIAA;  - MHPG levels were only significantly correlated with DOPAC levels;  - No significant differences in CSF NA levels between individuals previously treated vs untreated. | The limited number of untreated individuals might have affected the results concerning treated vs untreated individuals. | 1 |
| ^11^ | MHPG | n.i | n.i | 22 | n.s | AD | n.s | Withdrawal | n.s | HPLC ED | -Correlation between anomalous performance on the attentional focusing test and reduced CSF levels of MHPG. - Males had significantly higher levels of CSF MHPG. |  | 1 |
| ^43^ | NA, MHPG | 12 | 41.5 | n.i | n.i | CONTR | n.s | n.s | n.s | HPLC ED | - CSF MHPG levels were found to be increased in Huntington’s disease clinical group compared to controls. | CSF of control subjects was obtained from individuals subjected to myelography with a suspected diagnosisof lumbar disc herniation without blockade of CSF circulation, confirmed after examination, or spontaneous CSF nasal fistula, not confirmed after examination. CSF nasal fistula was not confirmed, at neuroradiologic examination, in any of the three subjects; two control subjects had normal myelography | 1 |
| ^44^ | MHPG | n.i | n.i | 17,  10,  5,  10,  10,  13 | 62,  64,  68,  55,  60,  57 | AD mild  AD moderate  AD severe,  PD mild,  PD moderate,  PD severe | AD: functional impairment in performance of daily activi¬ ties and partly on the ability to take neuropsychologic tests; PD: H&Y | Withdrawal | n.s | HPLC | - No differences in CSF MHPG between clinical groups at baseline;  - CSF MHPG levels did not correlate significantly with age. |  | 1 |
| ^45^ | NA, MHPG | n.i | n.i | 12 | 74.9 | AD | Ferm's and  GBS | Medication not discountinued | DSM III and NINCDS-ADRDA | HPLC | - CSF MHPG levels were significantly lower during selegiline treatment than during placebo;  - CSF NA levels did not differ significantly between selegiline and placebo. | -The individuals were hospitalized throughout the trial.  - Any previous medication or physiotherapy for concurrent diseases was continued during the trial. | 1 |
| ^46^ | MHPG | n.i | n.i | 64 | 73.1 | AD | MMSE | n.s | NINCDS-ADRDA | HPLC | - The addition of MHPG to Ab42, t-tau, and p-tau improves the discrimination of DLB from AD.  - Lower CSF MHPG levels in DBL clinical group compared with the AD clinical group. | Internal controls were included in all enzyme-linked immunoassays (ELISAs) to control for interassay variation.  *No other information was reported about the control group, therefore, this study was not included in dataset 2 despite reporting a control group.* | 1 |
| ^47^ | MHPG | n.i | n.i | 111 | 63.01 | AD | MMSE | n.s | Based on recommendations from the National Institute on Aging Alzheimer’s Association workgroups | HPLC | - Greater levels of CSF MHPG were associated with higher p-tau, independently of amyloidosis;  - Greater levels of CSF MHPG were associated with memory deficits via tau and inflammation-associated amyloidosis pathology;  - Neuropsychiatric symptoms were directly associated with CSF MHPG, and this relationship was partially mediated by tau pathology. |  | 1 |
| ^48^ | MHPG | 10 | 68.8 | 10 | n.s | CONTR  AD | n.s | n.s | NINCDS-ADRDA, DSM-IV-TR, LP and neurocognitive assessment including MMSE | RP-UHPLC-ED | - CSF MHPG levels differed across CSF fractions. | *CSF MHPG data form AD group was not reported, therefore, this study was not included in dataset 2.* | 1 |
| ^49^ | NA, MHPG | n.i | n.i | 12,  14 | 11.4,7.9 | PD,  PD with depression | H&Y | untreated | CURS, DSM III | HPLC | - No differences in CSF MHPG levels between individuals with PD with and without depression. |  | 1 |
| ^50^ | MHPG | 17 | 63.9 | n.i | n.i | CONTR | n.s | n.s | n.s | HPLC/GC | Individuals diagnosed with Korsakoff´s diseases have higher CSF MHPG levels compared to controls. | Some data analysed by HPLC some with GC. | 1 |
| ^51^ | MHPG | 19 | From 54 to 83 | n.i | n.i | CONTR | n.s | n.s | n.s | HPLC ED | - CSF MHPG did not differ between individuals with normal pressure hydrocephalus and controls. | In the control group were included 15 asymptomatic individuals with positive treponemal tests in the serum but negative CSF findings and normal MMSE, as well as four individuals with a few non-specific T2 lesions on MRI and normal neurological examination, CSF analysis and visual evoked potentials | 1 |
| ^52^ | MHPG | n.s | n.s | 5,  16 | n.s | AD,  PD | n.s | Treated and untreated | n.s | HPLC | - Untrated individuals in the PD clincal group shows higher level of CSF MHPG compared to controls. | *This study was not included in dataset 2 despite reporting a control group because the control group was composed by individuals with other neurological problems, whose influence on noradrenergic levels cannot be ruled out.* | 1 |
| ^53^ | MHPG | n.i | n.i | 14 | 65.3 | PD with depression | H&Y, HDRS, MADRS, UPDRS. | Withdrawal | The United Kingdom PD Society Brain Bank, DSM III - IV | MF | -Lower baseline CSF MHPG levels in the PD clinical group with concurrent depression, compared to the group with solely depression.  -Decreased CSF MHPG levels in individuals with depression after citalopram, that did not happen for individuals with PD and with depression. |  | 1 |
| ^54^ | NA, MHPG | n.i | n.i | 12 | 82.2 | SDAT | GBS | None of them were taking neuroleptics and antidepressant drugs. | DMS III | HPLC ED | - No differences in CSF MHPG levels between individuals with AD and individuals with schizophrenia. | The individuals with dementia had been in the hospital for at least 5 years and had progressive mental deterioration and no evident signs of vascular disorders or arteriosclerotic risk factors. | 1 |
| ^55^ | NA | 10 | 71 | 10 | 70 | AD mild | MMSE | Untreated + Withdrawal | n.s | Single isotope radioenzymatic method | - No significant differences in CSF NA levels between clinical group and controls;  - Higher CSF NA concentrations in older controls and in individuals with AD compared to young controls. |  | 1 |
| ^56^ | NA | 33,  14 | 25,  65 | n.i | n.i | HC | n.s | n.s | n.s | RM | - CSF NA was significantly higher in older men than in young men;   - -The suppression of CSF NA by clonidine was significantly greater in young men than older men. | *Only the control group of older adults was included in the data of dataset 1.* | 1 |
| ^57^ | NA,  MHPG | 10,  10 | 27,  60 | n.i | n.i | HC | n.s | n.s | n.s | HPLC ED | - No difference in CSF NA and CSF MHPG levels between older and younger individuals | *Only the control group of older adults was included in the data of dataset 1.* | 1 |
| ^58^ | MHPG | n.i | n.i | 87,  11 | 77.5,  74.6 | PD without NMS, PD-NMS | H&Y | treated | Presence of two or more of the following symptoms: tremor, rigidity, akinesia and postural instability, and responsiveness to L-DOPA. | HPLC ED | - No differences in CSF MHPG levels and 5-HIAA levels between groups;  -The PD NMS clinical group showed worse parkinsonian disability than the PD group without NMS. |  | 1 |
| ^59^ | NA | n.i | n.i | 202 | 80.4 | AD | MMSE, GDetS , HDS | Treated and untreated | NINCDS-ADRDA, DSM IV | HPLC with a fluorescence detector | - No significant differences in CSF NA levels were found when comparing untreated individuals with individuals receiving L-DOPA. |  | 1 |
| ^60^ | MHPG | n.i | n.i | 6 | 56.3 | PD | n.s | treated | n.s | HPLC | - L-Threo-DOPS was effective for akinesia in 7 of 10 individuals with PD. |  | 1 |
| ^61^ | NA, MHPG | 8,7 | n.s | 6 | n.s | PD | n.s | Treated, untreated | n.s | HPLC ED | *Evaluation of analysis methods. No useful findings for the purpose of this review (CSF noradrenergic levels in clinical group or control group) had been reported or discussed* | *Only PD clinical group was included in the dataset 1, as no information of age of the control group was reported.* | 1 |
| **Ref.** | **Controls** | | | **PD or AD dementia group** | | | | |  | **Reference & UoM** | **Finding** | **Considerations** | **Dataset** |
|  | **Tracer** | **n** | **Age (M)** | **n** | **Age (M)** | **Clinical stage** | **Severity**  **Based on** | **Treatment** | **Pathology assessment** |  |  |  |  |
| ^62^ | MeNER | 10 | 65.7 | 15 | 64.8 | PD mild | H&Y | Overnight withdrawal | n.s. | Caudate and Occipital cortex (BPnd) | - Individuals diagnosed with PD had lower MeNER binding in all regions compared to controls. -The reduction was significant in the nucleus ruber and thalamus. |  | 1,2 |
|  | MeNER | 12 | 67.3 | 14 | 65.4 | PD without RBD (RBD-), mild | H&Y | Withdrawal | MDS | Occipital cortex (DVRs) | - PD RBD+ group showed significantly lower levels compared to controls in   - Thalamus; - Hypothalamus; - Raphe nucleus;   - PD RBD- group showed significantly lower levels compared to controls in   - Thalamus; - Hypothalamus; - Raphe nucleus; - LC; - DR; - MR;   - And reduced levels compared to RBD+ in   - Thalamus; - Hypothalamus; - LC; - DR; - MR;   -Reduced MeNER binding in PD with RBD correlated with EEG slowing, cognitive performances and hypotension. | RBD | 1,2 |
| ^13^ | MeNER | 12 | 67.3 | 30 | 66.6 | PD | H&Y | Withdrawal | MDS | Thalamus/Caudate | -Lower MeNER binding in the primary motor cortex and primary sensory cortex in individuals in the PD group compared to controls.  -Individuals with PD in more severe stages have more pronounced reduction in MeNER binding. |  | 1,2 |

**Supplementary Table 2. Meta-analysis results (*dataset 2*).** The results are reported before the exclusion of outliers (1), after exclusion based on confidence intervals (2) and after the Leave-One-Out method and the Graphic Display of Heterogeneity (GOSH) plots (3). Between-studies heterogeneity (I-squared) drops consistently in every group after exclusion of studies indicated by the GOSH plot, indicating a decrease in heterogeneity between studies. In the meta-analysis plot, we reported the results after excluding both outliers (2) and influential studies (3).

In the AD group reduced the between-studies heterogeneity (I-squared) from 45.73% to 11.99% for NA, and from 78.92% to 67.87% for MHPG, leading to a change in the p-value from 0.090 to 0.042 and from 0.825 to 0.778, with the exclusion of k= 2 and k= 3 studies, respectively

In the PD group we observe that the exclusion of outliers reduced the between-studies heterogeneity (I-squared) from 45.05% to 0.00% for NA and from 82.81% to 0.00% for MHPG, leading to a change of the p-value from 0.019 to 0.012 and from 0.733 to 0.006 respectively, with the exclusion of k= 2 and k= 1 studies. ADD = Alzheimer’s disease dementia; I2 = I-squared; k = number of studies; lower = lower bound of the 95% confidence interval; MHPG = 3-Methoxy-4-hydroxyphenylglycol; NA = Noradrenaline; PD = Parkinson’s disease dementia; se = standard error; upper = higher bound of the 95% confidence interval. Significance levels are indicated by asterisks (*p<0.05, ** p<0.01, ***p<0.001)

| **Group** | **k** | **Hedge’s g** | **se** | **lower** | **upper** | **t** | **p-value** | **I2** | **lower I2** | **upper I2** | **p-value I2** | **Out**  **1** | **Out**  **2** | **Out**  **3** |
| --- | --- | --- | --- | --- | --- | --- | --- | --- | --- | --- | --- | --- | --- | --- |
| NA_ADD 1 | 11 | -0.06 | 0.25 | -0.62 | 0.50 | -0.23 | 0.825 | 78.92% | 0.63 | 0.88 | 0.00 |  |  |  |
| NA_ADD 2 | 11 | -0.06 | 0.25 | -0.62 | 0.50 | -0.23 | 0.825 | 78.92% | 0.63 | 0.88 | 0.00 |  |  |  |
| NA_ADD 3 | 9 | -0.06 | 0.20 | -0.53 | 0.41 | -0.29 | 0.778 | 67.87% | 0.35 | 0.84 | 0.00 | ^32^ | ^36^ |  |
| NA_PD 1 | 10 | -0.51 | 0.18 | -0.91 | -0.11 | -2.87 | 0.019 * | 45.05% | 0.00 | 0.74 | 0.06 |  |  |  |
| NA_PD 2 | 9 | -0.38 | 0.11 | -0.63 | -0.13 | -3.47 | 0.008 ** | 0.00% | 0.00 | 0.55 | 0.62 | ^26^ |  |  |
| NA_PD 3 | 8 | -0.26 | 0.08 | -0.44 | -0.08 | -3.36 | 0.012 * | 0.00% | 0.00 | 0.00 | 0.96 | ^26^ | ^17^ |  |
| MHPG_ADD 1 | 13 | 0.25 | 0.14 | -0.05 | 0.55 | 1.84 | 0.090 | 45.73% | 0.00 | 0.72 | 0.04 |  |  |  |
| MHPG_ADD 2 | 13 | 0.25 | 0.14 | -0.05 | 0.55 | 1.84 | 0.090 | 45.73% | 0.00 | 0.72 | 0.04 |  |  |  |
| MHPG_ADD 3 | 10 | 0.29 | 0.12 | 0.01 | 0.57 | 2.36 | 0.042 * | 11.99% | 0.00 | 0.53 | 0.33 | ^19^ | ^17^ | ^36^ |
| MHPG_PD 1 | 14 | -0.07 | 0.19 | -0.49 | 0.35 | -0.35 | 0.733 | 82.81% | 0.72 | 0.89 | 0.00 |  |  |  |
| MHPG_PD 2 | 13 | -0.27 | 0.08 | -0.44 | -0.09 | -3.37 | 0.006 ** | 0.00% | 0.00 | 0.18 | 0.90 | ^32^ |  |  |
| MHPG_PD 3 | 13 | -0.27 | 0.08 | -0.44 | -0.09 | -3.37 | 0.006 ** | 0.00% | 0.00 | 0.18 | 0.90 | ^32^ |  |  |

**Supplementary** **Table 3. Weighted averages (*dataset 1*).** Weighted average means of the groups were calculated using the single studies sample

sizes as weight. The cross (†) indicates the measures after the exclusion of the outliers.

ADD = Alzheimer’s disease dementia; CONTR = controls; MHPG = 3-Methoxy-4-hydroxyphenylglycol; NA = noradrenaline; PD = Parkinson’s disease dementia; SD = Standard deviation

| **Group** | **Weighted mean** | **Weighted SD** | **N° of articles** | **N° of participants** |
| --- | --- | --- | --- | --- |
| CONTR_MHPG | 11.10 | 6.59 | 22 | 402 |
| PD_MHPG | 16.28 | 17.85 | 28 | 514 |
| ADD_MHPG | 10.63 | 8.18 | 25 | 590 |
| CONTR_NA | 419.13 | 479.95 | 16 | 319 |
| PD_NA | 400.80 | 378.96 | 17 | 229 |
| ADD_NA | 361.03 | 153.53 | 16 | 626 |
| CONTR_MHPG † | 8.66 | 2.14 | 20 | 349 |
| PD_MHPG † | 10.46 | 5.11 | 27 | 461 |
| ADD_MHPG † | 8.19 | 2.43 | 23 | 538 |
| CONTR_NA † | 193.17 | 74.59 | 14 | 260 |
| PD_NA † | 205.49 | 90.88 | 16 | 179 |
| ADD_NA † | 344.28 | 144.70 | 15 | 585 |

**Supplementary** **Table 4. Regressions on *dataset 1 (fullmodels)*.** ADD: Alzheimer’s disease dementia; CONTR: Controls; csfvol: Volume of the CSF sample; Df: Degrees of freedom; k: number of studies; MHPG = 3-Methoxy-4-ydroxyphenylglycol; n = sample size; NA = noradrenaline; PD = Parkinson’s disease dementia; Pr(>F) = p-value associated with the F statistic; Res.SE = residual standard error; ypd = years post diagnosis.

Significance levels are indicated by asterisks (*p<0.05, ** p<0.01, ***p<0.001)

| **Group** | **k** | **Variables excluded** | **Model**  **(full model)** | **Df** | **Res.SE** | | **Adjusted R-squared** | **F** | **p-value** |
| --- | --- | --- | --- | --- | --- | --- | --- | --- | --- |
| ***~ n + method + csfvol + age + severity + ypd*** | | | | | | | | | |
| ADD_MHPG | 25 | ypd,  severity | ~ n + method + csfvol+ age | 20 | | 7.603 | -0.03381 | 0.8038 | 0.5371 |
| ADD_NA | 16 | ypd | ~ n + method + csfvol + age + severity | 10 | | 134.2 | 0.2156 | 1.824 | 0.1957 |
| PD_MHPG | 28 | ypd,  severity | ~ n + method + csfvol + age | 20 | | 11.02 | 0.1512 | 1.687 | 0.1693 |
| PD_NA | 18 | ypd,  severity | ~ n+ method + csfvol + age | 12 | | 124.8 | 0.1794 | 1.743 | 0.1993 |
| ***~ n + method + csfvol + age*** | | | | | | | | | |
| CONTR_ MHPG | 22 | ypd,  severity as not applicable to controls | ~ n + method + csfvol + age | 15 | | 5.916 | -0.0504 | 0.832 | 0.5635 |
| CONTR_NA | 16 | ypd,  severity as not applicable to controls | ~ n + method + csfvol + age | 11 | | 428.8 | 0.01406 | 1.053 | 0.4239 |
| ***~ n + method + csfvol + group + age + severity + ypd*** | | | | | | | | | |
| MHPG | 75 | ypd,  severity | ~ n + method + csfvol + group | 64 | | 8.561 | 0.08473 | 1.685 | 0.1037 |
| NA | 50 | ypd,  severity | ~ n + method + csfvol + group | 42 | | 262.3 | 0.0642 | 1.48 | 0.2006 |
| ***Additional interaction terms included:*** *~ n + method + csfvol + age* severity + age* ypd* | | | | | | | | | |
| PD_MHPG | 28 | ypd,  severity | n + method + csfvol + age * severity + age * ypd | 16 | | 11.94 | 0.002696 | 1.007 | 0.4816 |
| PD_NA | 18 | ypd,  severity | n + method + csfvol + age * severity + age * ypd | 8 | | 115.4 | 0.2988 | 1.805 | 0.2088 |

**Supplementary** **Table 5. Stepwise regressions on *dataset 1*.** The table shows that a significant reduced model (~ n + age) was found for PD MHPG (p=0.018), MHPG (model: ~ n + csfvol + age; p=0.003) and NA (model: ~ n + age; p=0.032).

ADD = Alzheimer’s disease dementia; CONTR = controls; csfvol = volume of the CSF sample; Df = degrees of freedom; k = number of studies;

MHPG = 3-Methoxy-4-ydroxyphenylglycol; n = sample size; NA = noradrenaline; PD = Parkinson’s disease dementia; Pr(>F) = p-value associated with the F statistic; Res.SE = residual standard error; ypd = years post diagnosis. Significance levels are indicated by asterisks (*p<0.05, ** p<0.01, ***p<0.001)

| **Group** | **k** | **Variables excluded** | **Full Model** | **Reduced Model** | **Df** | **Res.SE** | | **Adjusted R-squared** | **F** | **p-value** |
| --- | --- | --- | --- | --- | --- | --- | --- | --- | --- | --- |
| ***~ n + method + csfvol + age + severity + ypd*** | | | | | | | | | | |
| ADD_MHPG | 25 | ypd,  severity | ~ n + method + csfvol+ age | ~ csfvol | 23 | | 7.274 | 0.05396 | 2.369 | 0.137 |
| ADD_NA | 16 | ypd | ~ n + method + csfvol + age + severity | ~ n + age + severity | 12 | | 125.4 | 0.3147 | 3.296 | 0.058 |
| PD_MHPG | 28 | ypd,  severity | ~ n + method + csfvol + age | ~ n + age | 25 | | 10.58 | 0.218 | 4.764 | 0.018 * |
| PD_NA | 18 | ypd,  severity | ~ n+ method + csfvol + age | ~ method + age | 14 | | 117.5 | 0.2733 | 3.131 | 0.059 |
| ***~ n + method + csfvol + age*** | | | | | | | | | | |
| CONTR_ MHPG | 22 | ypd,  severity as not applicable to controls | ~ n + method + csfvol + age | ~ n | 20 | | 5.625 | 0.05028 | 2.112 | 0.162 |
| CONTR_NA | 16 | ypd,  severity as not applicable to controls | ~ n + method + csfvol + age | - | 15 | | 431.9 | - | - | - |
| ***~ n + method + csfvol + group + age + severity + ypd*** | | | | | | | | | | |
| MHPG | 75 | ypd,  severity | ~ n + method + csfvol + group | ~ n + csfvol + age | 71 | | 8.288 | 0.1423 | 5.091 | 0.003 ** |
| NA | 50 | ypd,  severity | ~ n + method + csfvol + group | ~ n+ age | 47 | | 257.3 | 0.09926 | 3.7 | 0.032 * |

**Supplementary** **Table 6. ANOVA analysis. Comparison between full models and reduced models**.

Csfvol = Volume of the CSF sample; Df = Degrees of freedom; MHPG = 3-Methoxy-4-ydroxyphenylglycol; n = sample size; NA = noradrenaline; PD = Parkinson’s disease dementia; Pr(>F) = p-value associated with the F statistic; Res.Df = residual degrees of freedom for each model; RSS = residual sum of squares for each model; Sum of Sq = sum of squares; ypd = years post diagnosis.

Significance levels are indicated by asterisks (*p<0.05, ** p<0.01, ***p<0.001)

| **Group** | | **Models compared**  **(Full vs Reduced)** | **Adjusted R-squared** | **Res.Df** | | **RSS** | **Df** | **Sum of Sq** | **F** | **Pr(<F)** |
| --- | --- | --- | --- | --- | --- | --- | --- | --- | --- | --- |
| AD_MHPG | ~ n + method + csfvol+ age  ~ csfvol | | -0.034  0.054 | | 20  23 | 1156.3  1216.8 | -3 | -60.546 | 0.3491 | 0.7902 |
| AD_NA | ~ n + method + csfvol + age + severity  ~ n + age + severity | | 0.2156  0.3147 | | 10  12 | 180040  188755 | -2 | -8714.2 | 0.242 | 0.7895 |
| PD_MHPG | ~ n + method + csfvol + age  ~ n + age | | 0.151  0.218 | | 20  25 | 2428.8  2796.7 | -5 | -367.95 | 0.606 | 0.6962 |
| PD_NA | ~ n+ method + csfvol + age  ~ method + age | | 0.1794  0.2733 | | 12  14 | 186971  193183 | -2 | -6212 | 0.1993 | 0.8219 |
| CONTR_MHPG | ~ n + method + csfvol + age  ~ n | | -0.050  0.0503 | | 15  20 | 524.92  632.81 | -5 | -107.89 | 0.6166 | 0.6892 |
| CONTR_NA | ~ n + method + csfvol + age  - | | 0.0141  - | | 11  15 | 2022711  2797584 | -4 | -774873 | 1.0535 | 0.4239 |
| MHPG | ~ n + method + csfvol + group  ~ n + csfvol + age | | 0.0847  0.1423 | | 64  71 | 4691  4877 | -7 | -189.01 | 0.3625 | 0.9206 |
| NA | ~ n + method + csfvol + group  ~ n+ age | | 0.0642  0.0992 | | 42  47 | 2889736  3112622 | -5 | -222886 | 0.6479 | 0.6646 |

**Supplementary** **Table 7. ANOVA analysis. Comparison between full models and full models with additional interaction terms (*age*severity* and *age*ypd*)**.

csfvol = volume of the csf sample; Df = degrees of freedom; MHPG = 3-Methoxy-4-ydroxyphenylglycol; n = sample size; NA = noradrenaline; PD = Parkinson’s disease dementia; Pr(>F) = p-value associated with the F statistic; Res.Df = residual degrees of freedom for each model; RSS = residual sum of squares for each model; Sum of Sq = sum of squares; ypd = years post diagnosis.

Significance levels are indicated by asterisks (*p<0.05, ** p<0.01, ***p<0.001)

| **Group** | **Models compared**  **(Full vs Full with additional interaction terms)** | | **Adjusted R-squared** | **Res.Df** | | **RSS** | **Df** | **Sum of Sq** | **F** | **Pr(<F)** |
| --- | --- | --- | --- | --- | --- | --- | --- | --- | --- | --- |
| PD_MHPG | | ~ n + method + csfvol + age  ~ n + method + csfvol + age * severity + age * ypd | 0.218  0.002696 | | 20  16 | 2428.8  2282.8 | 4 | 145.93 | 0.2557 | 0.9019 |
| PD_NA | | ~ n+ method + csfvol + age  ~ n + method + csfvol + age * severity + age * ypd | 0.2733  0.2988 | | 12  18 | 186971  106511 | 4 | 80460 | 1.5108 | 0.2866 |

## Supplementary References

References of the Supplementary Information:

1. Abdo WF, Bloem BR, Van Geel WJ, Esselink RAJ, Verbeek MM. CSF neurofilament light chain and tau differentiate multiple system atrophy from Parkinson’s disease. *Neurobiology of Aging*. 2007;28(5):742-747. doi:10.1016/j.neurobiolaging.2006.03.010

2. Chia LG, Cheng LJ, Chuo LJ, Cheng FC, Cu JS. Studies of dementia, depression, electrophysiology and cerebrospinal fluid monoamine metabolites in patients with Parkinson’s disease. *Journal of the Neurological Sciences*. 1995;133(1-2):73-78. doi:10.1016/0022-510X(95)00146-S

3. Raskind MA, Peskind ER, Holmes C, Goldstein DS. Patterns of cerebrospinal fluid catechols support increased central noradrenergic responsiveness in aging and Alzheimer’s disease. *Biological Psychiatry*. 1999;46(6):756-765. doi:10.1016/S0006-3223(99)00008-6

4. Tohgi H, Abe T, Takahashi S, Takahashi J, Ueno M, Nozaki Y. Effect of a synthetic norepinephrine precursor, l-threo-3,4-dihydroxyphenylserine on the total norepinephrine concentration in the cerebrospinal fluid of parkinsonian patients. *Neuroscience Letters*. 1990;116(1-2):194-197. doi:10.1016/0304-3940(90)90409-3

5. Mayeux R, Stern Y, Cote L, Williams JBW. Altered serotonin metabolism in depressed patients with Parkinson’s disease. Published online 1984:6.

6. Abdo WF, De Jong D, Hendriks JCM, et al. Cerebrospinal fluid analysis differentiates multiple system atrophy from Parkinson’s disease. *Mov Disord*. 2004;19(5):571-579. doi:10.1002/mds.10714

7. Chia LG, Cheng FC, Kuo JS. Monoamines and their metabotites in plasma and lumbar cerebrospinal fluid of Chinese patients with Parkinson’s disease. Published online 1993. doi:10.1016/0022-510x(93)90316-q

8. Peskind ER. Effects of Alzheimer’s Disease and Normal Aging on Cerebrospinal Fluid Norepinephrine Responses to Yohimbine and Clonidine. *Arch Gen Psychiatry*. 1995;52(9):774. doi:10.1001/archpsyc.1995.03950210068012

9. Tohgi H, Abe T, Takahashi S. The effects of L-threo-3,4-dihydroxyphenylserine on the total norepinephrine and dopamine concentrations in the cerebrospinal fluid and freezing gait in parkinsonian patients. *J Neural Transm Gen Sect*. 1993;5(1):27-34. doi:10.1007/BF02260912

10. Mayeux. Clinical and biochemical features of depression in Parkinson’s disease. *AJP*. 1986;143(6):756-759. doi:10.1176/ajp.143.6.756

11. Freed DM, Corkin S, Growdon JH, Nissen MJ. Selective attention in Alzheimer’s disease: Characterizing cognitive subgroups of patients. *Neuropsychologia*. 1989;27(3):325-339. doi:10.1016/0028-3932(89)90022-5

12. Nahimi A, Sommerauer M, Kinnerup MB, et al. Noradrenergic Deficits in Parkinson Disease Imaged with ^11^ C-MeNER. *J Nucl Med*. 2018;59(4):659-664. doi:10.2967/jnumed.117.190975

13. Sommerauer M, Hansen AK, Parbo P, et al. Decreased noradrenaline transporter density in the motor cortex of Parkinson’s disease patients: Cortical Noradrenaline Transporter. *Mov Disord*. 2018;33(6):1006-1010. doi:10.1002/mds.27411

14. Sommerauer M, Fedorova TD, Hansen AK, et al. Evaluation of the noradrenergic system in Parkinson’s disease: an 11C-MeNER PET and neuromelanin MRI study. *Brain*. 2018;141(2):496-504. doi:10.1093/brain/awx348

15. Rücker G, Cates CJ, Schwarzer G. Methods for including information from multi-arm trials in pairwise meta-analysis: Multi-arm trials in pairwise meta-analysis. *Res Syn Meth*. 2017;8(4):392-403. doi:10.1002/jrsm.1259

16. Martignoni E, Bono G, Blandini F, Sinforiani E, Merlo P, Nappi G. Monoamines and related metabolite levels in the cerebrospinal fluid of patients with dementia of Alzheimer type. Influence of treatment with L-deprenyl. *J Neural Transm Gen Sect*. 1991;3(1):15-25. doi:10.1007/BF02251133

17. Martignoni E, Blandini F, Petraglia F, Pacchetti C, Bono G, Nappi G. Cerebrospinal fluid norepinephrine, 3-methoxy-4-hydroxyphenylglycol and neuropeptide Y levels in Parkinson’s disease, multiple system atrophy and dementia of the Alzheimer type. *J Neural Transm Gen Sect*. 1992;4(3):191-205. doi:10.1007/BF02260903

18. Stefani A, Olivola E, Liguori C, et al. Catecholamine-Based Treatment in AD Patients: Expectations and Delusions. *Front Aging Neurosci*. 2015;7. doi:10.3389/fnagi.2015.00067

19. Tohgi H, Ueno M, Abe T, Takahashi S, Nozaki Y. Concentration of monoamines and their metabolites in the cerebrospinal fluid from patients with senile dementia of the Alzheimer type and vascular dementia of the Binswanger type. *J Neural Transm Gen Sect*. 1992;4(1):69-77. doi:10.1007/BF02257623

20. Milanov I, Bogdanova D. Pain and tension-type headache: areview of the possible pathophysiologicalmechanisms. *J Headache Pain*. 2004;5(1):4-11. doi:10.1007/s10194-004-0061-1

21. Palmer AM, Sims NR, Bowen DM, et al. Monoamine metabolite concentrations in lumbar cerebrospinal fluid of patients with histologically verified Alzheimer’s dementia. *Journal of Neurology, Neurosurgery & Psychiatry*. 1984;47(5):481-484. doi:10.1136/jnnp.47.5.481

22. Turkka JT, Juujärvi KK, Myllylä VV. Correlation of Autonomic Dysfunction to CSF Concentrations of Noradrenaline and 3-Methoxy-4-Hydroxyphenylglycol in Parkinson’s Disease. *Eur Neurol*. 1987;26(1):29-34. doi:10.1159/000116308

23. Janssens J, Vermeiren Y, Fransen E, et al. Cerebrospinal fluid and serum MHPG improve Alzheimer’s disease versus dementia with Lewy bodies differential diagnosis. *Alzheimer’s & Dementia: Diagnosis, Assessment & Disease Monitoring*. 2018;10:172-181. doi:10.1016/j.dadm.2018.01.002

24. Viechtbauer W. Conducting Meta-Analyses in *R* with the **metafor** Package. *J Stat Soft*. 2010;36(3). doi:10.18637/jss.v036.i03

25. Olkin I, Dahabreh IJ, Trikalinos TA. GOSH - a graphical display of study heterogeneity: ALL-SUBSETS META-ANALYSIS FOR HETEROGENEITY. *Res Syn Meth*. 2012;3(3):214-223. doi:10.1002/jrsm.1053

26. Cerroni R, Liguori C, Stefani A, et al. Increased Noradrenaline as an Additional Cerebrospinal Fluid Biomarker in PSP-Like Parkinsonism. *Front Aging Neurosci*. 2020;12:126. doi:10.3389/fnagi.2020.00126

27. Davidson DL, Yates CM, Mawdsley C, Pullar IA, Wilson H. CSF studies on the relationship between dopamine and 5-hydroxytryptamine in Parkinsonism and other movement disorders. *Journal of Neurology, Neurosurgery & Psychiatry*. 1977;40(12):1136-1141. doi:10.1136/jnnp.40.12.1136

28. Elrod. Effects of Alzheimer’s disease severity on cerebrospinal fluid norepinephrine concentration. *AJP*. 1997;154(1):25-30. doi:10.1176/ajp.154.1.25

29. Bråne G, Gottfries CG, Blennow K, et al. Monoamine metabolites in cerebrospinal fluid and behavioral ratings in patients with early and late onset of Alzheimer dementia. *Alzheimer Dis Assoc Disord*. 1989;3(3):148-156. doi:10.1097/00002093-198903030-00004

30. Hartikainen P, Reinikainen KJ, Soininen H, Sirviö J, Soikkeli R, Riekkinen PJ. Neurochemical markers in the cerebrospinal fluid of patients with Alzheimer’s disease, Parkinson’s disease and amyotrophic lateral sclerosis and normal controls. *J Neural Transm Gen Sect*. 1992;4(1):53-68. doi:10.1007/BF02257622

31. Ichikawa N. Study on monoamine metabolite contents of cerebrospinal fluid in patients with neurodegenerative diseases. *Tohoku J Exp Med*. 1986;150(4):435-446. doi:10.1620/tjem.150.435

32. Janssens J, Vermeiren Y, Fransen E, et al. Cerebrospinal fluid and serum MHPG improve Alzheimer’s disease versus dementia with Lewy bodies differential diagnosis. *Alzheimer’s & Dementia: Diagnosis, Assessment & Disease Monitoring*. 2018;10:172-181. doi:10.1016/j.dadm.2018.01.002

33. Kay AD, Milstien S, Kaufman S, et al. Cerebrospinal Fluid Biopterin Is Decreased in Alzheimer’s Disease. *Archives of Neurology*. 1986;43(10):996-999. doi:10.1001/archneur.1986.00520100018008

34. Mann DMA. The locus coeruleus and its possible role in ageing and degenerative disease of the human central nervous system. *Mechanisms of Ageing and Development*. 1983;23(1):73-94. doi:10.1016/0047-6374(83)90100-8

35. Peskind ER, Wingerson D, Pascualy M, et al. Oral physostigmine in alzheimer’s disease: Effects on norepinephrine and vasopressin in cerebrospinal fluid and plasma. *Biological Psychiatry*. 1995;38(8):532-538. doi:10.1016/0006-3223(94)00377-F

36. Raskind MA. Norepinephrine and MHPG Levels in CSF and Plasma in Alzheimer’s Disease. *Arch Gen Psychiatry*. 1984;41(4):343. doi:10.1001/archpsyc.1984.01790150033006

37. Sunderland T, Tariot PN, Cohen RM, et al. Dose-dependent effects of deprenyl on CSF monoamine metabolites in patients with Alzheimer’s disease. *Psychopharmacology*. 1987;91(3):293-296. doi:10.1007/BF00518180

38. Tohgi H, Abe T, Takahashi S, Takahashi J, Ueno M, Nozaki Y. Effect of a synthetic norepinephrine precursor, l-threo-3,4-dihydroxyphenylserine on the total norepinephrine concentration in the cerebrospinal fluid of parkinsonian patients. *Neuroscience Letters*. 1990;116(1-2):194-197. doi:10.1016/0304-3940(90)90409-3

39. Sheline YI, Miller K, Bardgett ME, Csernansky JG. Higher Cerebrospinal Fluid MHPG in Subjects With Dementia of the Alzheimer Type: Relationship With Cognitive Dysfunction. *The American Journal of Geriatric Psychiatry*. 1998;6(2):155-161. doi:10.1097/00019442-199805000-00009

40. Andersen AD, Blaabjerg M, Binzer M, et al. Cerebrospinal fluid levels of catecholamines and its metabolites in Parkinson’s disease: effect of l -DOPA treatment and changes in levodopa-induced dyskinesia. *J Neurochem*. 2017;141(4):614-625. doi:10.1111/jnc.13997

41. Corona GL, Cucchi ML, Frattini P, et al. Clinical and biochemical responses to therapy in Alzheimer’s disease and multi-infarct dementia. *Eur Arch Psychiatr Neurol Sci*. 1989;239(2):79-86. doi:10.1007/BF01759579

42. Engelborghs S, Vloeberghs E, Le Bastard N, et al. The dopaminergic neurotransmitter system is associated with aggression and agitation in frontotemporal dementia. *Neurochemistry International*. 2008;52(6):1052-1060. doi:10.1016/j.neuint.2007.10.018

43. Garrett MC, Soares-da-Silva P. Increased Cerebrospinal Fluid Dopamine and 3,4-Dihydroxyphenylacetic Acid Levels in Huntington’s Disease: Evidence for an Overactive Dopaminergic Brain Transmission. *J Neurochem*. 1992;58(1):101-106. doi:10.1111/j.1471-4159.1992.tb09283.x

44. Gibson CJ, Logue M, Growdon JH. CSF Monoamine Metabolite Levels in Alzheimer’s and Parkinson’s Disease. *Archives of Neurology*. 1985;42(5):489-492. doi:10.1001/archneur.1985.04060050091016

45. Heinonen EH, Savijärvi M, Kotila M, Hajba A, Scheinin M. Effects of monoamine oxidase inhibition by selegiline on concentrations of noradrenaline and monoamine metabolites in CSF of patients with Alzheimer’s disease. *J Neural Transm Gen Sect*. 1993;5(3):193-202. doi:10.1007/BF02257674

46. Herbert MK, Aerts MB, Kuiperij HB, et al. Addition of MHPG to Alzheimer’s disease biomarkers improves differentiation of dementia with Lewy bodies from Alzheimer’s disease but not other dementias. *Alzheimer’s & Dementia*. 2014;10(4):448-455.e2. doi:10.1016/j.jalz.2013.05.1775

47. Jacobs HIL, Riphagen JM, Ramakers IHGB, Verhey FRJ. Alzheimer’s disease pathology: pathways between central norepinephrine activity, memory, and neuropsychiatric symptoms. *Mol Psychiatry*. Published online May 28, 2019. doi:10.1038/s41380-019-0437-x

48. Janssens J, Atmosoerodjo SD, Vermeiren Y, Absalom AR, den Daas I, De Deyn PP. Sampling issues of cerebrospinal fluid and plasma monoamines: Investigation of the circadian rhythm and rostrocaudal concentration gradient. *Neurochemistry International*. 2019;128:154-162. doi:10.1016/j.neuint.2019.04.015

49. Kuhn W, Müller Th, Gerlach M, et al. Depression in Parkinson’s disease: biogenic amines in CSF of  “de novo” patients. *J Neural Transmission*. 1996;103(12):1441-1445. doi:10.1007/BF01271258

50. Mair RG, Mcentee WJ, Zatorre RJ. Monoamine activity correlates with psychometric deficits in Korsakoff’s disease. *Behavioural Brain Research*. 1985;15(3):247-254. doi:10.1016/0166-4328(85)90179-2

51. Markianos M, Lafazanos S, Koutsis G, Sfagos C, Seretis A. CSF neurotransmitter metabolites and neuropsychiatric symptomatology in patients with normal pressure hydrocephalus. *Clinical Neurology and Neurosurgery*. 2009;111(3):231-234. doi:10.1016/j.clineuro.2008.10.001

52. Mena MA, Aguado EG, de Yebenes JG. Monoamine metabolites in human cerebrospinal fluid. HPLC/ED method. *Acta Neurologica Scandinavica*. 1984;69(4):218-225. doi:https://doi.org/10.1111/j.1600-0404.1984.tb07804.x

53. Pålhagen S, Qi H, Mårtensson B, Wålinder J, Granérus AK, Svenningsson P. Monoamines, BDNF, IL-6 and corticosterone in CSF in patients with Parkinson’s disease and major depression. *J Neurol*. 2010;257(4):524-532. doi:10.1007/s00415-009-5353-6

54. Parnetti L, Gottfries J, Karlsson I, Långström G, Gottfries CG, Svennerholm L. Monoamines and their metabolites in cerebrospinal fluid of patients with senile dementia of Alzheimer type using high performance liquid chromatography and gas chromatography-mass spectrometry. *Acta Psychiatr Scand*. 1987;75(5):542-548. doi:10.1111/j.1600-0447.1987.tb02831.x

55. Peskind ER. Effects of Alzheimer’s Disease and Normal Aging on Cerebrospinal Fluid Norepinephrine Responses to Yohimbine and Clonidine. *Arch Gen Psychiatry*. 1995;52(9):774. doi:10.1001/archpsyc.1995.03950210068012

56. Raskind MA, Peskind ER, Veith RC, Beard JC, Gumbrecht G, Halter JB. Increased Plasma and Cerebrospinal Fluid Norepinephrine in Older Men: Differential Suppression by Clonidine*. *The Journal of Clinical Endocrinology & Metabolism*. 1988;66(2):438-443. doi:10.1210/jcem-66-2-438

57. Schapiro MB, Kay AD, May C, et al. Cerebrospinal fluid monoamines in Down’s syndrome adults at different ages. *Journal of Intellectual Disability Research*. 2008;31(3):259-269. doi:10.1111/j.1365-2788.1987.tb01369.x

58. Ueda M, Hamamoto M, Nagayama H, et al. Susceptibility to neuroleptic malignant syndrome in Parkinson’s disease. *Neurology*. 1999;52(4):777-777. doi:10.1212/WNL.52.4.777

59. Vermeiren Y, Le Bastard N, Van Hemelrijck A, Drinkenburg WH, Engelborghs S, De Deyn PP. Behavioral correlates of cerebrospinal fluid amino acid and biogenic amine neurotransmitter alterations in dementia. *Alzheimer’s & Dementia*. 2013;9(5):488-498. doi:10.1016/j.jalz.2012.06.010

60. Yamamoto M, Ogawa N, Ujike H. Effect of l-Threo-3,4-dihydroxyphenylserine chronic administration on cerebrospinal fluid and plasma free 3-methoxy-4-hydroxy-phenylglycol concentration in patients with Parkinson’s disease. *Journal of the Neurological Sciences*. 1986;73(1):39-44. doi:10.1016/0022-510X(86)90061-4

61. Zhang W, Xie Y, Gu J, et al. Liquid chromatography with amperometric detection at a nano crystalline Ce-doped lead dioxide film modified electrode for determination of (R)-Salsolinol, (R)-N-methylsalsolinol and monoamine neurotransmitters in Parkinsonian patients’ cerebrospinal fluid. *Analyst*. 2004;129(3):229. doi:10.1039/b314277a

62. Nahimi A, Kinnerup MB, Sommerauer M, Gjedde A, Borghammer P. Molecular Imaging of the Noradrenergic System in Idiopathic Parkinson’s Disease. In: *International Review of Neurobiology*. Vol 141. Elsevier; 2018:251-274. doi:10.1016/bs.irn.2018.07.028
